# Multiscale DCE-BOLD MRI reveals vascular uncoupling patterns for IDH genotyping in adult glioma: A prospective habitat-based study

**DOI:** 10.1016/j.neurot.2026.e01114

**Published:** 2026-10-30

**Authors:** Dandan Song, Yutian Wang, Borui Li, Yan Liu, Peixu Guo, Yaqin Mi, Haiyuan Qu, Siru Nie, Yueluan Jiang, Yang Song, Guoguang Fan, Zixuan Lin, Miao Chang

**Affiliations:** a Department of Radiology, the First Hospital of China Medical University, Shenyang, China; b Zhejiang Key Laboratory of Intelligent Sensing Technology and Advanced Medical Instrument and Key Laboratory for Biomedical Engineering of Ministry of Education, College of Biomedical Engineering & Instrument Science, Zhejiang University, Hangzhou, China; c Department of Pathology, the First Hospital of China Medical University, Shenyang, China; d MR Research Collaboration, Siemens Healthineers, Beijing, China; e MR Research Collaboration, Siemens Healthineers, Shanghai, China

**Keywords:** Glioma, DCE-MRI, rs-BOLD-fMRI, Habitat imaging, IDH mutation

## Abstract

Isocitrate dehydrogenase (IDH)-wildtype gliomas exhibit heterogeneous vascular dysfunction, but whole-tumor analyses may obscure regional variation in vascular permeability and functional reserve. We developed a multiscale habitat framework integrating dynamic contrast-enhanced magnetic resonance imaging (DCE-MRI)-derived volume transfer constant (*K*^trans^) and resting-state blood oxygen level-dependent functional MRI (rs-BOLD-fMRI)-derived relative cerebrovascular reactivity (*r*CVR), including a 10-mm peritumoral margin, for noninvasive IDH genotyping. This prospective single-center cohort included 132 patients (52 IDH-mutant and 80 IDH-wildtype), divided chronologically into training (n = 92) and internal test (n = 40) cohorts. Gaussian mixture modeling partitioned the continuous joint *K*^trans^-*r*CVR distribution into three overlapping imaging-defined intratumoral subregions; the anatomically defined peritumoral region was analyzed separately. The Habitat-Peri10mm model yielded the highest internal test area under the receiver operating characteristic curve (AUC) point estimate of 0.914 (95% confidence interval, 0.831–0.997), compared with 0.854 for the Habitat model and 0.753 for the Whole-tumor model. Pairwise DeLong tests were nonsignificant (p = 0.173 and 0.054, respectively). A Subregion 2 *K*^trans^ first-order Entropy feature ranked highest in the SHapley Additive exPlanations (SHAP) analysis and correlated with Ki-67 (r = 0.57) and microvessel density (r = 0.27). The habitat-derived intratumoral heterogeneity score was higher in IDH-wildtype than IDH-mutant tumors (0.79 vs. 0.40, p < 0.001; AUC = 0.824), although grade imbalance limited IDH-specific interpretation. This DCE-BOLD multiscale habitat framework characterizes IDH-associated patterns of vascular heterogeneity and shows potential for noninvasive IDH genotyping.

## Introduction

Glioma, the most common and aggressive primary malignant brain tumor in adults, is characterized by profound therapeutic resistance and poor prognosis [1]. This aggressiveness is fundamentally rooted in a highly heterogeneous and dynamic tumor microenvironment (TME) [2], [3], [4], within which aberrant vascular architecture and regional hypoxia constitute critical drivers of malignancy [5], [6], [7], [8]. This vascular pathology—typified by microvascular proliferation and necrosis—is associated with isocitrate dehydrogenase (IDH) mutation status [9], [10]. Experimental studies have linked mutant IDH-derived 2-hydroxyglutarate (2-HG) to altered regulation of hypoxia-inducible factor-1alpha (HIF-1α) [11], while HIF-1α/vascular endothelial growth factor A (VEGF-A) signaling provides a biological context for angiogenesis in glioblastoma [12]. These observations motivate investigation of IDH-associated vascular patterns alongside variation related to tumor grade and histological context. Yet current IDH genotyping relies on invasive surgical sampling with inherent spatial bias—potentially missing the most aggressive subclones—and conventional structural magnetic resonance imaging (MRI) provides limited information about this functional heterogeneity. A noninvasive imaging strategy capable of resolving the spatial complexity of tumor vascular function is therefore urgently needed [13].

The volume transfer constant (*K*^trans^) from dynamic contrast-enhanced MRI (DCE-MRI) quantifies microvascular permeability and perfusion, reflecting structural endothelial integrity [14]. Relative cerebrovascular reactivity (*r*CVR), robustly measurable via resting-state blood oxygen level-dependent functional MRI (rs-BOLD-fMRI), assesses dynamic vasodilatory capacity—a functional reserve frequently impaired in gliomas due to neurovascular uncoupling [15], [16], [17], [18], [19], [20], [21]. Conventional CO_2_-inhalation challenge is the reference standard for cerebrovascular reactivity (CVR) mapping, but it requires specialized equipment and patient cooperation, limiting clinical translation. By contrast, rs-BOLD-fMRI-derived *r*CVR correlates strongly with CO_2_-inhalation CVR [16] and offers a noninvasive, task-free alternative that is readily integrated into routine protocols. *K*^trans^ and *r*CVR capture complementary dimensions of vascular health: *K*^trans^ reports on relatively static structural permeability, whereas *r*CVR reflects dynamic functional reserve. Their spatial interplay—where high permeability coexists with or diverges from preserved vascular reactivity—may reveal IDH-associated regional patterns of permeability–reactivity coupling [22], [23]. Joint spatial analysis of *K*^trans^ and *r*CVR offers an opportunity to characterize regional variation in vascular permeability and functional reserve within a habitat imaging framework.

Whole-tumor summaries may obscure regional variation in glioma imaging characteristics, while habitat imaging provides a spatial framework for characterizing this heterogeneity [24], [25], [26]. Previous studies have investigated dynamic susceptibility contrast (DSC) perfusion vascular habitats [24], combined perfusion–diffusion habitats based on DSC-derived relative cerebral blood volume (*r*CBV) and diffusion-weighted imaging (DWI)-derived apparent diffusion coefficient (ADC) [27], habitats combining DCE-MRI-derived *K*^trans^ and DWI-derived ADC maps [28], metabolic–hemodynamic habitats [29], and diffusion-derived vessel-density habitats [30] for glioma characterization, IDH mutation status prediction, or prognostic assessment. Building on these works, the present study focuses on the joint spatial distribution of *K*^trans^ and *r*CVR to examine permeability–reactivity patterns and extends the analysis to an anatomically defined 10-mm peritumoral compartment. This extension permits assessment of imaging variation beyond the tumor boundary, where infiltrative and reactive tissue changes may coexist [31], [32].

In this study, we developed a multiparametric habitat imaging framework integrating *K*^trans^ from DCE-MRI and *r*CVR from rs-BOLD-fMRI within an unsupervised clustering pipeline and extended the analysis to an anatomically defined 10-mm peritumoral region. We hypothesized that the joint spatial distribution of *K*^trans^ and *r*CVR would reveal relative permeability–reactivity patterns associated with IDH status, including an imaging pattern characterized by high permeability and impaired vascular reserve. We further explored associations between features derived from the imaging-defined intratumoral subregions and histopathological markers of proliferation and angiogenesis, including Ki-67 and microvessel density (MVD), and quantified intratumoral heterogeneity (ITH) in a prospective cohort of 132 patients with diffuse glioma.

## Methods

### Participants

A prospective study was conducted involving 132 patients with diffuse glioma who underwent surgery at the First Hospital of China Medical University between June 2024 and January 2026. All participants provided written informed consent, and the study was approved by the institutional review board (approval No.: 2024-957-2).

Inclusion criteria comprised: (1) radiologically confirmed solitary tumor within the cerebral hemispheres; (2) complete multimodal neuroimaging data, including structural MRI, such as pre-contrast T1-weighted imaging (T1WI) and T1-weighted contrast-enhanced imaging (T1CE), T2-weighted imaging (T2WI), T2-weighted fluid-attenuated inversion recovery (T2-FLAIR) sequences, rs-BOLD-fMRI, and DCE-MRI; (3) complete molecular profiling according to the 2021 World Health Organization (WHO) classification of central nervous system (CNS) tumors [9], encompassing IDH1/2 mutation status and 1p/19q chromosomal co-deletion status. Exclusion criteria included: (1) prior history of other brain tumors or treated/recurrent gliomas; (2) CNS lesions from infections, hemorrhage, or trauma; (3) cases without definitive formalin-fixed paraffin-embedded (FFPE) histopathology (e.g., frozen section only, or specimens sent externally without return of final pathology reports). The enrollment process is summarized in Supplementary Fig. S1.

### MRI acquisition and image processing

All participants underwent MRI examinations using 3.0-T scanners (MAGNETOM Vida and Prisma; Siemens Healthineers, Erlangen, Germany) equipped with a 64-channel phased-array head coil. The imaging protocol included routine non-contrast sequences, followed by rs-BOLD-fMRI, DCE-MRI, and T1CE, with all scans aligned to fully cover the tumor volume.

First, *r*CVR maps were generated from rs-BOLD-fMRI data following a published pipeline [16]. Briefly, preprocessing of rs-BOLD-fMRI data included motion correction (Statistical Parametric Mapping, SPM12), spatial smoothing (full width at half maximum, FWHM = 8 mm), linear detrending, and band-pass filtering (0.008–0.1 Hz). A reference BOLD time course was extracted from healthy brain regions contralateral to the tumor, verified by a neuroradiologist to be unaffected by the glioma. Voxel-wise general linear regression against this reference was performed to generate raw CVR maps, which were then normalized to the whole-brain mean to produce *r*CVR maps. Detailed parameters are provided in Supplementary Methods 1. DCE-MRI data were quantitatively processed to derive pharmacokinetic parameter maps (*K*^trans^, *V*_*e*_, *K*_*ep*_). Following motion correction and cross-sequence registration, a patient-specific arterial input function (AIF) was derived for each patient by placing a 3-voxel region of interest (ROI) in the ipsilateral internal carotid artery. The resulting curve was visually inspected to ensure a sharp bolus peak and no apparent partial-volume contamination. Quantitative maps were generated using the standard Tofts model with nonlinear least-squares fitting [33]. Scanning parameters and processing pipelines are detailed in the Supplementary Methods 1 and Supplementary Table S1.

All parametric maps were coregistered to the mean BOLD and peak-enhancement DCE references using rigid transformations (Supplementary Methods 2).

### Delineation of tumor and peritumoral volumes of interest

Delineation of whole-tumor and peritumoral volumes of interest (VOIs) was performed using ITK-SNAP (v3.8.0; http://www.itksnap.org) through manual slice-wise contouring. Contrast-enhancing tumors were annotated on axial T1CE, while non-enhancing tumors were delineated on T2-FLAIR images. The resultant VOIs were coregistered to DCE-derived *K*^trans^ and BOLD-derived *r*CVR maps. Peritumoral extension was generated by a 10-mm radial expansion of the whole-tumor VOI using the ROI Morphology module in nnFeature Explorer (nnFAE, v0.2.5; https://archive.ismrm.org/2023/2895.html), an extension of the FeAture Explorer (FAE) platform. To prevent anatomical overlap, spatial constraints were applied as follows: if the expanded VOI intersected critical structures (e.g., cranial boundaries, ventricular systems, basal cisterns, or major cerebral vasculature), expert-guided slice-wise refinement was performed (Fig. 1). A rigorous multi-tier quality control protocol was implemented to reduce errors from manual VOI delineation. See Supplementary Methods 3 for details.

**Fig. 1:**
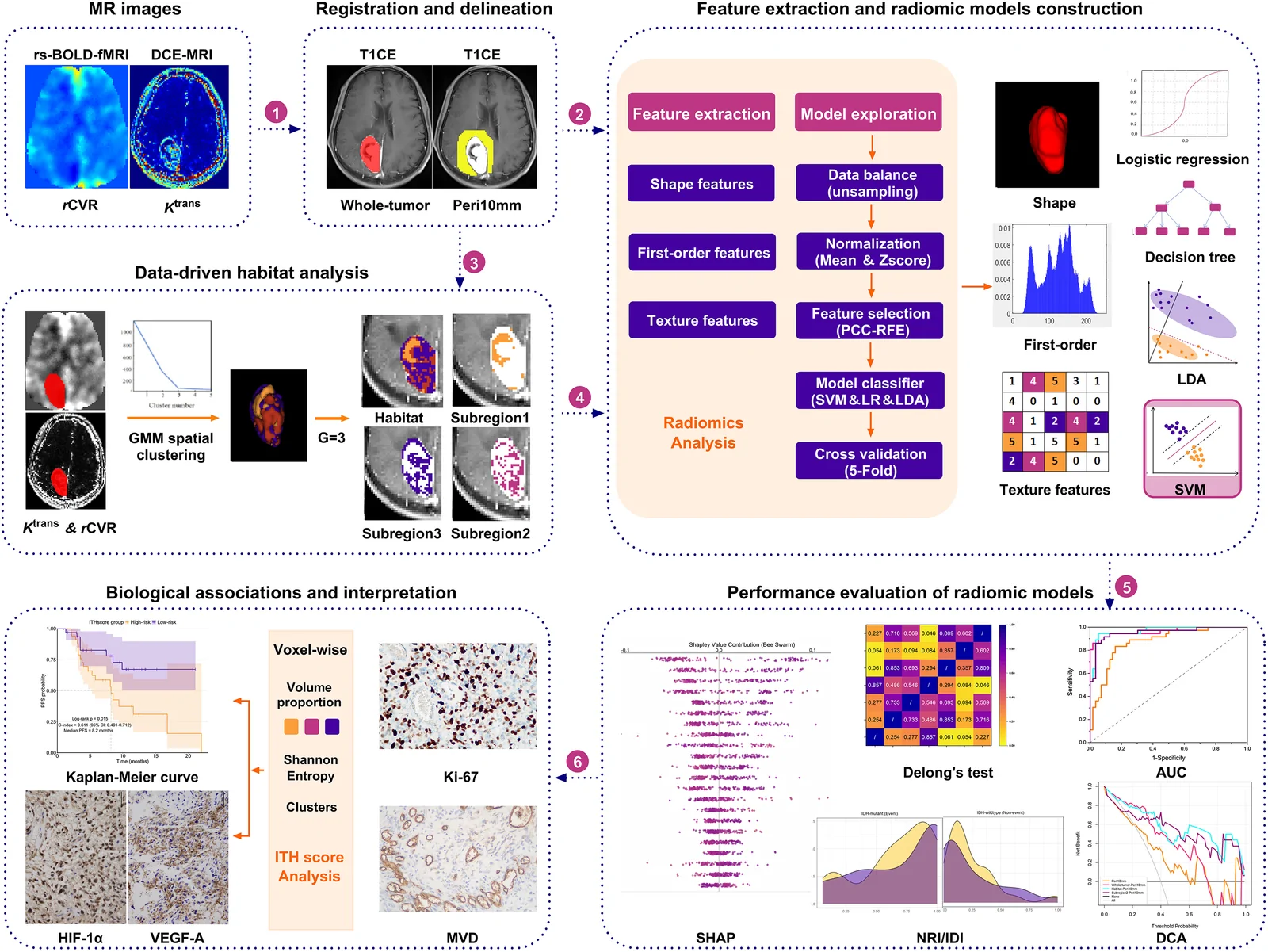
**Workflow of the DCE and BOLD-driven habitat analysis for IDH genotyping in glioma patients.***Abbreviations*: DCE-MRI, dynamic contrast-enhanced magnetic resonance imaging; rs-BOLD-fMRI, resting-state blood oxygen level-dependent functional magnetic resonance imaging; IDH, isocitrate dehydrogenase; *K*^trans^, volume transfer constant; *r*CVR, relative cerebrovascular reactivity; T1CE, T1-weighted contrast-enhanced imaging; Peri10mm, peritumoral 10 mm region; GMM, gaussian mixture model; PCC, pearson correlation coefficient; RFE, recursive feature elimination; SVM, support vector machine; LDA, linear discriminant analysis; LR, logistic regression; ITH, intratumoral heterogeneity; AUC, area under the curve; DCA, decision curve analysis; SHAP, shapley additive explanations; NRI, net reclassification improvement; IDI, integrated discrimination improvement; HIF-1α, hypoxia-inducible factor-1 alpha; VEGF, vascular endothelial growth factor; MVD, microvessel density.

### Spatial habitat profiling driven by coupled *K*^trans^ and *r*CVR

Habitat analysis was performed using nnFAE (v0.2.5; Python 3.11.4). The respective roles of nnFAE and FAE in this study are summarized in Supplementary Table S2. The input data comprised preprocessed multiparametric maps—specifically, *K*^trans^ and *r*CVR—along with their corresponding whole-tumor VOI masks in Neuroimaging Informatics Technology Initiative (NIfTI) format. Before Gaussian mixture model (GMM) clustering, both *K*^trans^ and *r*CVR maps were normalized (zero mean, unit variance). Pooled intratumoral voxels from the training cohort were used to fit the three-component GMM selected according to the Akaike information criterion (AIC). The fitted GMM parameters were subsequently fixed and applied to the internal test cohort without refitting. Bootstrap stability analysis was performed within the training cohort using 100 resamples, with more than 70% of voxels showing an assignment frequency greater than 0.7 (Supplementary Results 1).

### Radiomic modeling of tumor and peritumoral regions

Radiomic features were extracted using the open-source FeAture Explorer platform (FAE v0.6.0, https://github.com/salan668/FAE), incorporating PyRadiomics (v3.0) [34]. For each parametric map (*K*^trans^ and *r*CVR), 107 features were extracted separately from the whole-tumor VOI, each of the three intratumoral subregions, and the 10-mm peritumoral (Peri10mm) VOI, comprising 14 shape-based, 18 first-order, and 75 texture features. Because shape features were derived from the same regional segmentation mask for both parametric maps, only one set of 14 shape features was retained per region. Each region therefore contributed 200 candidate features before subsequent preprocessing and feature selection: 14 shape features and 93 first-order and texture features from each parametric map. Image intensities were discretized using a fixed bin width of 25 for texture feature calculation. The volume of each intratumoral subregion was also recorded. Patients were divided chronologically into training (70%) and internal test (30%) cohorts according to enrollment date.

Regional models were developed separately using the feature sets from the whole-tumor VOI, each intratumoral subregion, and the Peri10mm VOI. For the Habitat model, features retained from the three individual subregion models were concatenated to form a combined candidate feature set, which underwent further feature selection and classifier fitting within the training cohort.

Further details of radiomic preprocessing, feature selection, model development, and internal sensitivity analyses are provided in Supplementary Methods 4.

### Performance evaluation of tumor and peritumoral radiomics

Radiomic model performance was evaluated using receiver operating characteristic (ROC) curve analysis. Reported metrics included the area under the curve (AUC), accuracy, sensitivity, specificity, positive predictive value, and negative predictive value. The classification threshold for each model was selected by maximizing the Youden index. Additional internal sensitivity analyses included leave-one-out cross-validation (LOOCV) and permutation testing with 1000 label permutations for each evaluated radiomic model within the training cohort. Details are provided in Supplementary Methods 4.

### Multiscale radiomics for IDH genotyping

Multiscale models were constructed by combining selected intratumoral and peritumoral radiomic features. For the Habitat-Peri10mm model, features retained from the three individual intratumoral subregion models and the Peri10mm model were concatenated to form a combined candidate feature set, followed by further feature selection and classifier fitting. The Whole-tumor-Peri10mm model combined selected whole-tumor and peritumoral features. Model development was performed within the training cohort, and the resulting models were evaluated in the internal test cohort.

### Comparative analysis of DCE-BOLD-based radiomic models

Single-scale (Habitat, Whole-tumor, and Peri10mm) and multiscale (Habitat-Peri10mm) models were compared using ROC analysis, with paired AUC differences assessed using DeLong’s test (MedCalc v15.6.1). Decision curve analysis (DCA) was used to evaluate potential net benefit across threshold probabilities of 10%–90%. Continuous net reclassification improvement (NRI) and integrated discrimination improvement (IDI) were calculated as secondary metrics to characterize differences in model predictions.

### Radscore and SHAP interpretability for the DCE-BOLD-based optimal model

Based on the final trained predictive model, a radiomic score (Radscore) was computed for each patient. This score was defined as the linear weighted sum of the selected radiomic feature values and their corresponding regression coefficients (β). Meanwhile, to interpret the decision-making mechanism of the optimal model, Shapley Additive exPlanations (SHAP; v0.41.0) was employed. Using 10,000 randomly sampled instances from the training set as the background distribution, SHAP quantified the contribution of each radiomic feature to the model’s predictive output, thereby providing model interpretability at both global and individual levels.

### Biological correlates of the DCE-BOLD-based optimal radiomic model

#### Characterization of imaging-defined subregions

To describe regional functional heterogeneity, mean *K*^trans^ and *r*CVR values were extracted from each of the three GMM-defined intratumoral subregions after voxel-wise spatial coregistration. The anatomically defined Peri10mm region was analyzed separately. Differences in these imaging measures between IDH subgroups were subsequently assessed.

#### Exploratory associations with histopathological and edema-related measures

To explore the potential biological relevance of the imaging-defined subregions, associations between selected subregion-derived radiomic features, including the integrated Radscore, and patient-level histopathological markers, including Ki-67 and MVD, were assessed using correlation analyses. Selected imaging features were also compared between patients with and without peritumoral edema. Because the histopathological measurements were not spatially matched to the imaging-defined subregions, these analyses were considered exploratory and were not interpreted as validation of subregion-specific biological identities. Standardized assessment methods for the relevant molecular markers (including the Ki-67 index, MVD, IDH genotyping) are detailed in the Supplementary Methods 5.

### Exploratory evaluation of the intratumoral heterogeneity (ITH) score

#### Quantification of intratumoral heterogeneity

The ITH score [35] was calculated from the GMM-derived intratumoral subregions using R (v4.5.2), with detailed methods provided in Supplementary Methods 6. To examine its association with IDH status, differences in the ITH score between IDH-mutant and IDH-wildtype groups were assessed using Student’s *t*-test or the Mann–Whitney *U* test, as appropriate. Additional stratification by epidermal growth factor receptor (EGFR) amplification status was performed as an exploratory subgroup analysis.

#### Exploratory analysis of progression-free survival (PFS)

Exploratory analyses of the association between the ITH score and PFS were performed using the available follow-up data. Detailed methods are provided in Supplementary Methods 7.

#### Exploratory correlations with HIF-1α and VEGF-A expression

Exploratory associations between the ITH score and HIF-1α and VEGF-A expression were assessed using Spearman rank correlation in a subset of 20 patients with IDH-wildtype gliomas. Details of the immunohistochemical assessment and quantification of marker expression are provided in Supplementary Methods 5.

### Statistical analysis

Statistical analyses were performed in SPSS (v26.0) and R. Continuous data are expressed as mean ± standard deviation (SD) or median with interquartile range (IQR), depending on normality (Shapiro-Wilk test). Group comparisons used Student’s *t*-test or Mann-Whitney *U* test; categorical variables were analyzed by the Chi-square test. Exploratory comparisons of regional *K*^trans^ and *r*CVR values across WHO grades 2, 3, and 4 within the IDH-mutant group were performed using one-way analysis of variance (ANOVA) or the Kruskal–Wallis test, as appropriate. Radiogenomic associations were assessed with Spearman’s correlation. Multiple comparisons were controlled using the Benjamini-Hochberg false discovery rate (FDR) procedure (q < 0.05). All tests were two-sided, with p < 0.05 considered statistically significant. Post-hoc power analysis (G∗Power 3.1) confirmed sufficient power (>80%) to detect a medium effect at α = 0.05, supporting the robustness of the findings.

## Results

### Demographics

The study cohort comprised 132 patients with histologically confirmed diffuse glioma (63 males and 69 females; mean age, 55.9 ± 12.4 years), including 52 patients with IDH-mutant tumors (WHO grades 2–4) and 80 with IDH-wildtype tumors (all WHO grade 4). Among the IDH-mutant tumors, 29 were WHO grade 2, 17 were grade 3, and six were grade 4 (Table 1). Patients were chronologically divided into a training cohort (n = 92, 70%) and a same-center internal temporal test cohort (n = 40, 30%). Detailed demographic and clinical characteristics are presented in Table 1 and Supplementary Table S3.

**Table 1:** Demographic and clinical characteristics by IDH status.

Characteristics	IDH-mutant (n = 52)	IDH-wildtype (n = 80)	*z*/*t* value	*p* value
**Age, years (IQR)**	49.50 (16.00)	62.50 (14.00)	−5.607	<0.001∗
**Gender, n (%)**				
Male	27 (20.45)	36 (27.27)	1.789	0.099
Female	25 (18.94)	44 (33.33)		
**Tumor type, n (%)**				
Astrocytoma	33 (25.00)	0 (0.00)	–	–
Oligodendroglioma	19 (14.39)	0 (0.00)		
Glioblastoma	0 (0.00)	80 (60.61)		
**Tumor location, n (%)**				
Frontal lobe	26 (19.70)	21 (15.91)	–	–
Temporal lobe	8 (6.06)	33 (25.00)		
Parietal lobe	9 (6.82)	12 (9.09)		
Occipital lobe	2 (1.51)	6 (4.54)		
Multilobar lobes	7 (5.30)	8 (6.06)		
**Peritumoral edema, n (%)**				
Yes	17 (12.88)	72 (54.55)	−6.839	<0.001∗
No	35 (26.51)	8 (6.06)		
**WHO grade, n (%)**				
2	29 (21.97)	0 (0.00)	–	–
3	17 (12.88)	0 (0.00)		
4	6 (4.54)	80 (60.61)		
**1p/19q status, n (%)**				
Codeleted	10 (7.57)	2 (1.51)	3.674	<0.001∗
Non-codeleted	9 (6.82)	29 (21.97)		
NA	33 (25.00)	49 (37.12)		
**MGMT status, n (%)**				
Methylated	18 (13.64)	15 (11.36)	3.324	0.002∗
Unmethylated	1 (0.76)	16 (12.12)		
NA	33 (25.00)	49 (37.12)		
**TERT status, n (%)**				
Mutant	10 (7.58)	23 (17.42)	−1.547	0.137
Wildtype	9 (6.82)	8 (6.06)		
NA	33 (25.00)	49 (37.12)		
**EGFR status, n (%)**				
Amplification	3 (2.27)	20 (15.15)	−3.322	<0.001∗
Non-amplification	16 (12.12)	11 (8.33)		
NA	33 (25.00)	49 (37.12)		
**Ki-67, % (IQR)**	10.00 (22.50)	35.00 (20.00)	−6.881	<0.001∗
**MVD, vessels/HPF**	22.72 (15.45)	43.60 (42.62)	−5.076	<0.001∗

*Note*: Data are presented as median (IQR), or n (%) as appropriate. A |z| > 1.96 corresponds to p < 0.05. ∗, represents statistical significance at p < 0.05.

*Abbreviations*: IQR, interquartile range; IDH, isocitrate dehydrogenase; WHO, world health organization; NA, no molecular results; 1p/19q, 1p/19q, the short arm of chromosome 1 (1p) and the long arm of chromosome 19 (19q); MGMT, O6-methylguanine-DNA methyltransferase; TERT, telomerase reverse transcriptase; EGFR, epidermal growth factor receptor; MVD, microvessel density (vessels/HPF); HPF, high-power field ( × 400).

### Spatial habitat profiling driven by coupled *K*^trans^ and *r*CVR

The three-component GMM solution had the lowest AIC among the evaluated solutions (AIC = 2.61 × 10^6^). The average silhouette score was 0.115, indicating substantial overlap between subregions in the joint *K*^trans^-*r*CVR feature space. The resulting subregions were therefore treated as imaging-defined partitions of a continuous distribution rather than as discrete biological entities. In 100 bootstrap resamples, more than 70% of voxels had an assignment frequency greater than 0.7, indicating relatively stable core assignments within the training cohort. Although three GMM components were applied across the training cohort, the number of observable subregions in individual tumors varied because some components were not represented in every tumor (Fig. 2a and b).

**Fig. 2:**
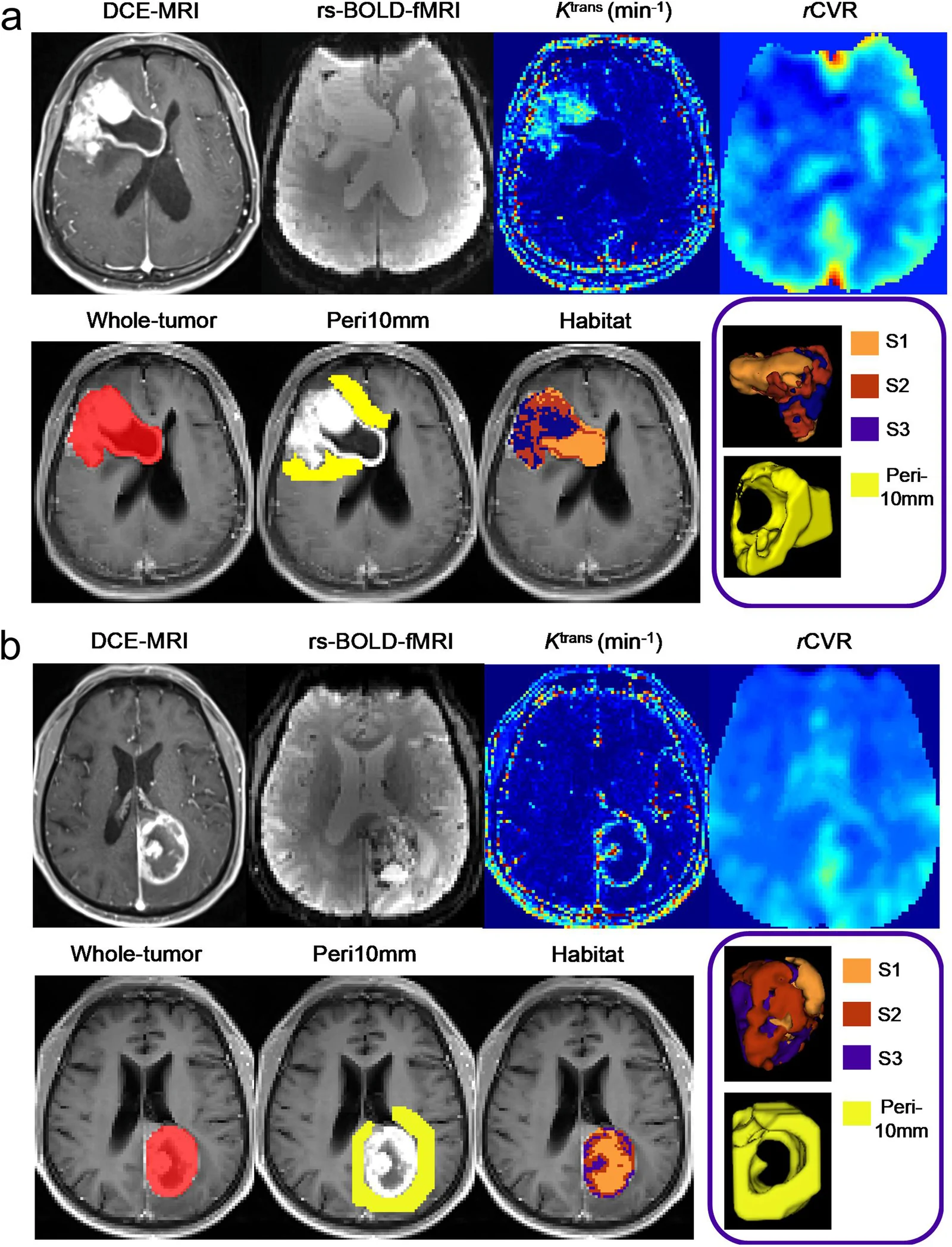
**Representative DCE-BOLD habitat imaging and peritumoral delineation in IDH-mutant versus wildtype gliomas.** (a) IDH-mutant oligodendroglioma (CNS WHO grade 3) in a 51-year-old female. (b) IDH-wildtype GBM in a 65-year-old male. *Abbreviations*: DCE-MRI, dynamic contrast-enhanced magnetic resonance imaging; rs-BOLD-fMRI, resting-state blood oxygen level-dependent functional magnetic resonance imaging; *K*^trans^, volume transfer constant; *r*CVR, relative cerebrovascular reactivity; Peri10mm, peritumoral 10 mm region; S1, subregion1; S2, subregion2; S3, subregion3; IDH, isocitrate dehydrogenase; CNS, central nervous system; WHO, world health organization; GBM, glioblastoma.

### Performance of intratumoral and peritumoral radiomic models

In the internal test cohort, the Habitat model, derived from three GMM-defined intratumoral subregions, yielded an AUC of 0.854 (95% confidence interval [CI], 0.721–0.988), compared with 0.753 (95% CI, 0.589–0.917) for the Whole-tumor model and 0.776 (95% CI, 0.614–0.938) for the Peri10mm model. The corresponding retained feature counts were 13, 24, and seven, respectively. Among the individual subregion models, Subregion 2 yielded the largest test AUC point estimate of 0.841 (95% CI, 0.714–0.969) using eight retained features (Table 2, Fig. 3 and Supplementary Table S4).

**Table 2:** Performance of intratumoral and peritumoral radiomic models based on *K*^trans^ -*r*CVR.

Radiomic models/Feature count	AUC	95% CI	Sensitivity	Specificity	ACC	PPV	NPV	Youden	Cohorts
**Intratumoral models/Feature count**									
Whole-tumor (24)	0.954	0.912 - 0.997	0.972	0.929	0.946	0.897	0.981	0.901	Training
	0.753	0.589 - 0.917	0.500	0.958	0.775	0.889	0.742	0.458	Test
***Habitat (13)***	***0.945***	0.901 - 0.989	0.861	0.929	0.902	0.886	0.912	0.790	Training
	***0.854***	0.721 - 0.988	0.813	0.917	0.875	0.867	0.880	0.729	Test
Subregion1 (4)	0.860	0.772 - 0.947	0.667	0.964	0.848	0.923	0.818	0.631	Training
	0.792	0.621 - 0.962	0.813	0.833	0.825	0.765	0.870	0.646	Test
*Subregion2 (8)*	0.942	0.894 - 0.990	0.889	0.875	0.880	0.821	0.924	0.764	Training
	0.841	0.714 - 0.969	0.875	0.708	0.775	0.667	0.895	0.583	Test
Subregion3 (26)	0.953	0.912 - 0.994	0.944	0.893	0.913	0.850	0.962	0.837	Training
	0.818	0.663 - 0.973	0.688	0.958	0.850	0.917	0.821	0.646	Test
**Peritumoral model/Feature count**									
Peri10mm (7)	0.871	0.796 - 0.945	0.833	0.839	0.837	0.769	0.887	0.673	Training
	0.776	0.614 - 0.938	0.750	0.833	0.800	0.750	0.833	0.583	Test
**Combined models/Feature count**									
Whole-tumor - Peri10mm (14)	0.967	0.927 - 1.000	0.944	0.946	0.946	0.919	0.964	0.891	Training
	0.867	0.758 - 0.976	0.813	0.833	0.825	0.765	0.870	0.646	Test
**Habitat - Peri10mm∗ (22)**	**0.967**	0.934 - 1.000	0.944	0.946	0.946	0.919	0.964	0.891	Training
	**0.914**	0.831 - 0.997	0.938	0.750	0.825	0.714	0.947	0.688	Test
Subregion2 - Peri10mm (15)	0.953	0.908 - 0.999	0.917	0.911	0.913	0.868	0.944	0.827	Training
	0.885	0.755 - 1.000	0.938	0.792	0.850	0.750	0.950	0.729	Test

*Note*: Numbers in parentheses indicate the number of features retained in the final models. Bold type with an asterisk (∗) identifies the model with the highest internal test AUC point estimate; italicized values identify the individual subregion model with the highest internal test AUC point estimate.

*Abbreviations*: AUC, area under the curve; 95% CI, 95% confidence interval; ACC, accuracy; PPV, positive predictive value; NPV, negative predictive value; DCE-MRI, dynamic contrast-enhanced magnetic resonance imaging; rs-BOLD-fMRI, resting state-blood oxygen level dependent-functional magnetic resonance imaging; *K*^trans^, volume transfer constant; *r*CVR, relative cerebrovascular reactivity; Peri10mm, peritumoral 10 mm region.

**Fig. 3:**
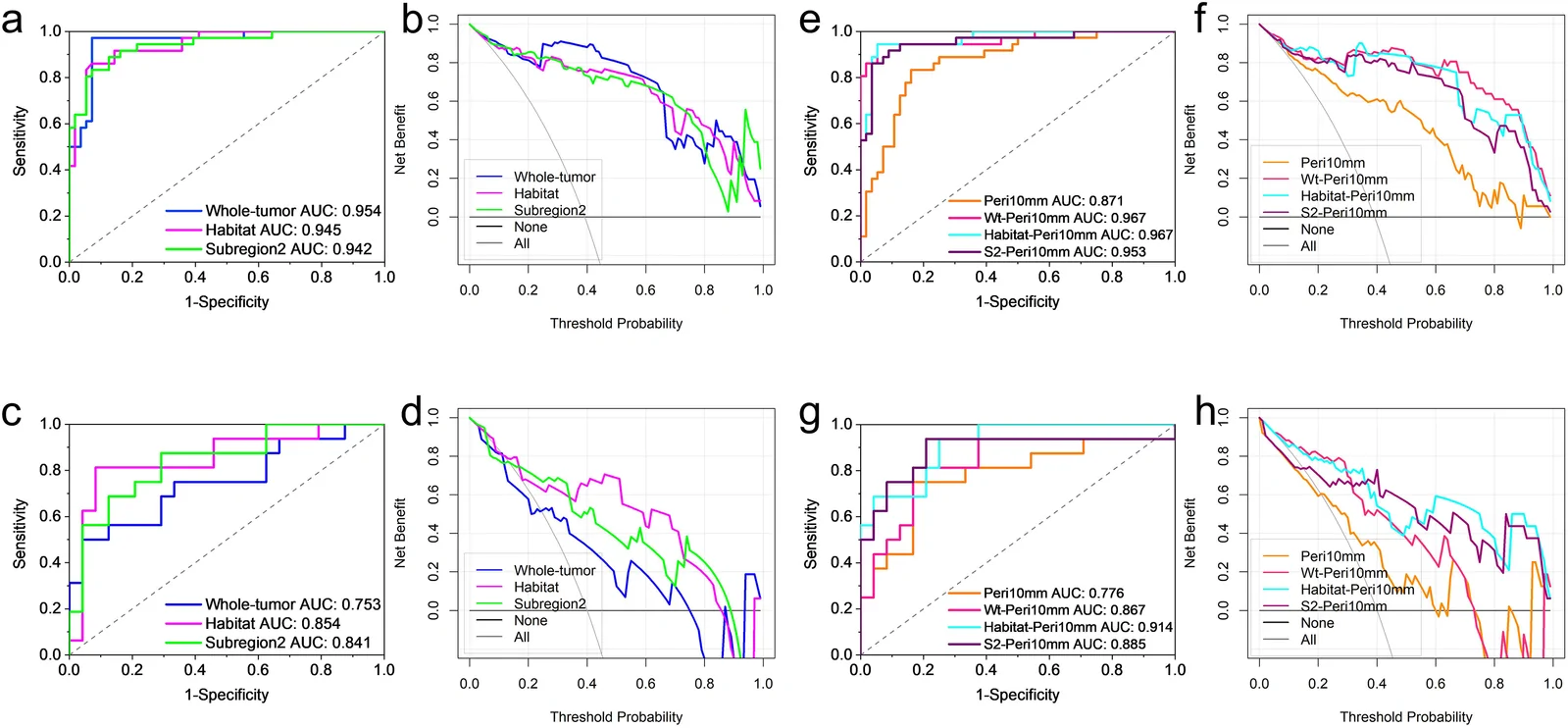
**ROC and decision curve analyses of radiomic models for IDH genotyping.** (a, b) Intratumoral radiomic model performance in training cohort; (a) ROC curve; (b) DCA. (c, d) Intratumoral radiomic model performance in test cohort; (c) ROC curve; (d) DCA. (e, f) Combined tumor and Peri10mm radiomic model performance in training cohort; (e) ROC curve; (f) DCA. (g, h) Combined tumor and Peri10mm radiomic model performance in test cohort; (g) ROC curve; (h) DCA. *Abbreviations*: IDH, isocitrate dehydrogenase; AUC, area under the curve; Wt, whole tumor; S2, subregion2; Peri10mm, peritumoral 10 mm region; ROC, receiver operating characteristic; DCA, decision curve analysis.

### Performance of multiscale radiomic models for IDH genotyping

The Habitat-Peri10mm model, which retained 22 features, yielded the largest internal test AUC point estimate of 0.914 (95% CI, 0.831–0.997). The paired AUC comparisons with the Whole-tumor, Habitat, and Peri10mm models were not statistically significant by DeLong testing (uncorrected p = 0.054, 0.173, and 0.084, respectively; Supplementary Fig. S2). The Whole-tumor-Peri10mm and Subregion 2-Peri10mm models yielded internal test AUCs of 0.867 (95% CI, 0.758–0.976) and 0.885 (95% CI, 0.755–1.000), respectively.

In additional internal sensitivity analyses, the Habitat-Peri10mm model yielded a LOOCV AUC of 0.858 (95% CI, 0.768–0.948) within the training cohort. The permutation test with 1000 permutations yielded p < 0.001 (Supplementary Results 3).

### Decision-curve and reclassification analyses

DCA demonstrated net clinical benefit for all models across a 10%–90% risk threshold range (Fig. 3). The reclassification results were not uniformly favorable. In the internal test cohort, nominally significant NRI values were reported for the Whole-tumor versus Habitat comparison (NRI = 0.396, p = 0.003), the Whole-tumor versus Habitat-Peri10mm comparison (NRI = 0.354, p = 0.046), the Peri10mm versus Habitat-Peri10mm comparison (NRI = 0.792, p = 0.007), and the Habitat versus Habitat-Peri10mm comparison (NRI = −0.250, p = 0.019). IDI was nominally significant only for the Peri10mm versus Habitat-Peri10mm comparison (IDI = 0.212, p = 0.024); the other IDI comparisons were not statistically significant (p = 0.142–0.423; Supplementary Table S5).

### Radscore and SHAP interpretation of the Habitat-Peri10mm model

Linear modeling and SHAP analysis provided complementary descriptions of feature contributions within the Habitat-Peri10mm model. In the linear model used to derive the Radscore, the *r*CVR gray-level size zone matrix (GLSZM) Small Area Emphasis feature from Subregion 2 had the most negative coefficient (β = −1.075), lower feature values were therefore associated with IDH-wildtype status (Supplementary Fig. S3). In the SHAP analysis, *K*^trans^ first-order Entropy from Subregion 2 showed the largest model-specific contribution to predictions (SHAP = 12.36; Fig. 4).

**Fig. 4:**
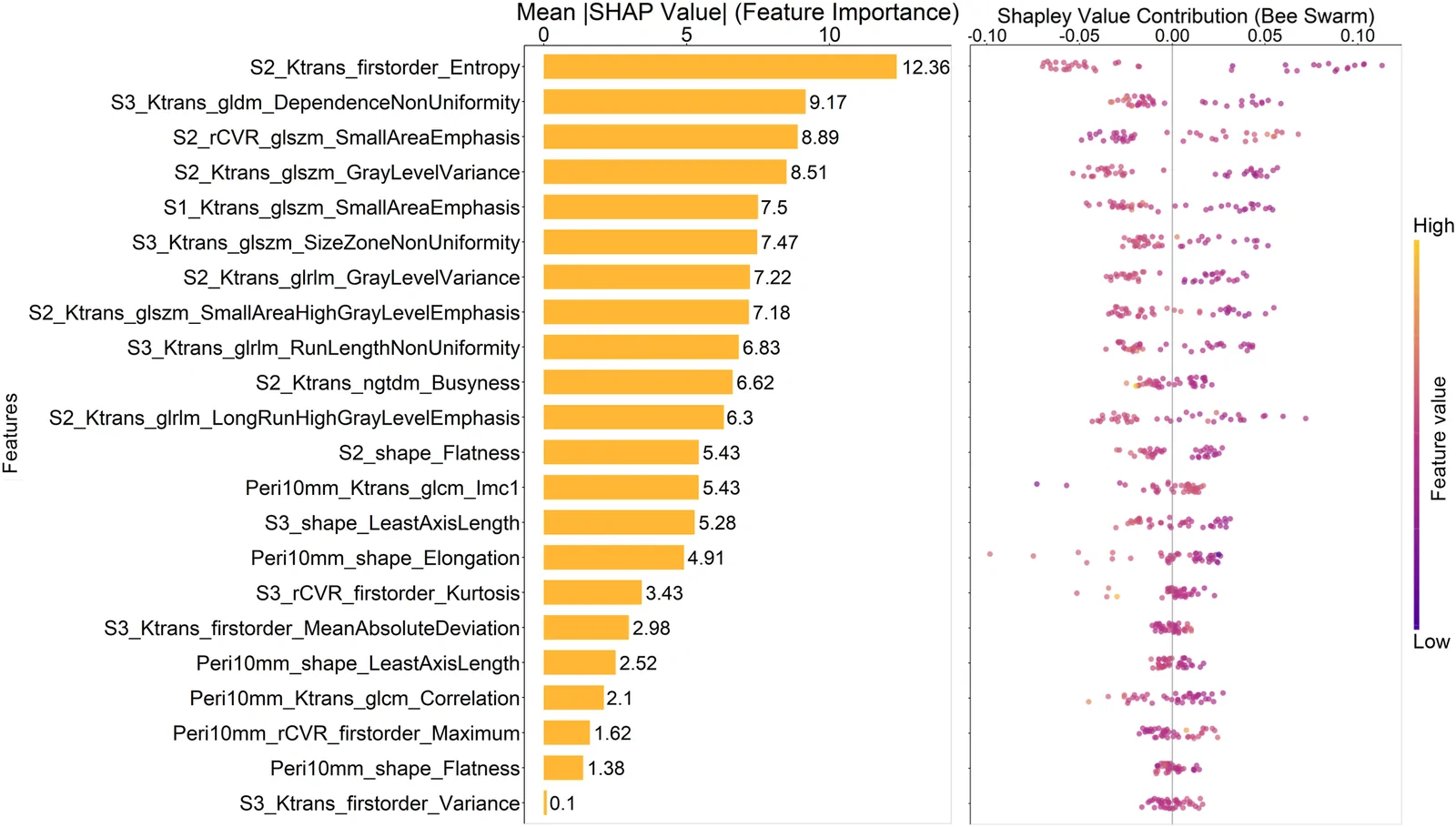
**SHAP interpretability for the optimal Habitat-Peri10mm radiomic model.***Abbreviations*: SHAP, shapley additive explanations; *K*^trans^, volume transfer constant; *r*CVR, relative cerebrovascular reactivity; S1, subregion1; S2, subregion2; S3, subregion3; Peri10mm, peritumoral 10 mm region; glszm, gray-level size zone matrix; glcm, gray-level co-occurrence matrix; ngtdm, neighborhood gray-tone difference matrix; gldm, gray-level dependence matrix; glrlm, gray-level run length matrix; Imc1, information measure of correlation1.

### Biological correlates of the Habitat-Peri10mm model

#### Characterization of relative imaging patterns

Mean *K*^trans^ and *r*CVR values were extracted from the three GMM-defined intratumoral subregions and the anatomically defined Peri10mm region to characterize regional functional heterogeneity. Across the cohort, these regions were descriptively categorized according to their relative permeability-reactivity patterns as low-permeability/low-reserve for Subregion 1, moderate-permeability/high-reserve for Subregion 2, high-permeability/low-reserve for Subregion 3, and low-permeability/high-reserve for the Peri10mm region (Table 3). These labels describe relative imaging patterns within the present cohort and do not represent validated biological thresholds.

**Table 3:** Regional imaging profiles based on mean *K*^trans^ and *r*CVR values.

Image-defined regions	*K*^trans^ (min^−1^)	*r*CVR	Relative permeability–reactivity profiles
**Tumor regions**			
Subregion1	12.22 ± 14.16	0.52 ± 0.44	Low-permeability/low-reserve
Subregion2	63.74 ± 14.16	0.57 ± 0.49	Moderate-permeability/high-reserve
Subregion3	276.27 ± 111.73	0.53 ± 0.50	High-permeability/low-reserve
**Peri10mm region**			
Peri10mm	31.17 ± 14.55	0.75 ± 0.26	Low-permeability/high-reserve

*Note*: Data are presented as mean ± SD. Profile labels describe relative imaging characteristics within this cohort.

*Abbreviations*: SD, standard deviation; *K*^trans^, volume transfer constant; *r*CVR, relative cerebrovascular reactivity; Peri10mm, peritumoral 10 mm region.

Intergroup comparisons demonstrated that only the *K*^trans^ value of Subregion2 differed significantly between IDH-mutant and IDH-wildtype groups (p < 0.001; Fig. 5a), while only the *r*CVR value of the Peri10mm region showed a significant between-group difference (p < 0.05; Fig. 5b) (Supplementary Table S6). In contrast, the *r*CVR value of Subregion2 did not reach statistical significance (p = 0.059), though a numerical difference was present. In exploratory comparisons within the IDH-mutant group, Subregion 2 *K*^trans^ differed across WHO grades 2–4 (p = 0.006; Supplementary Table S7).

**Fig. 5:**
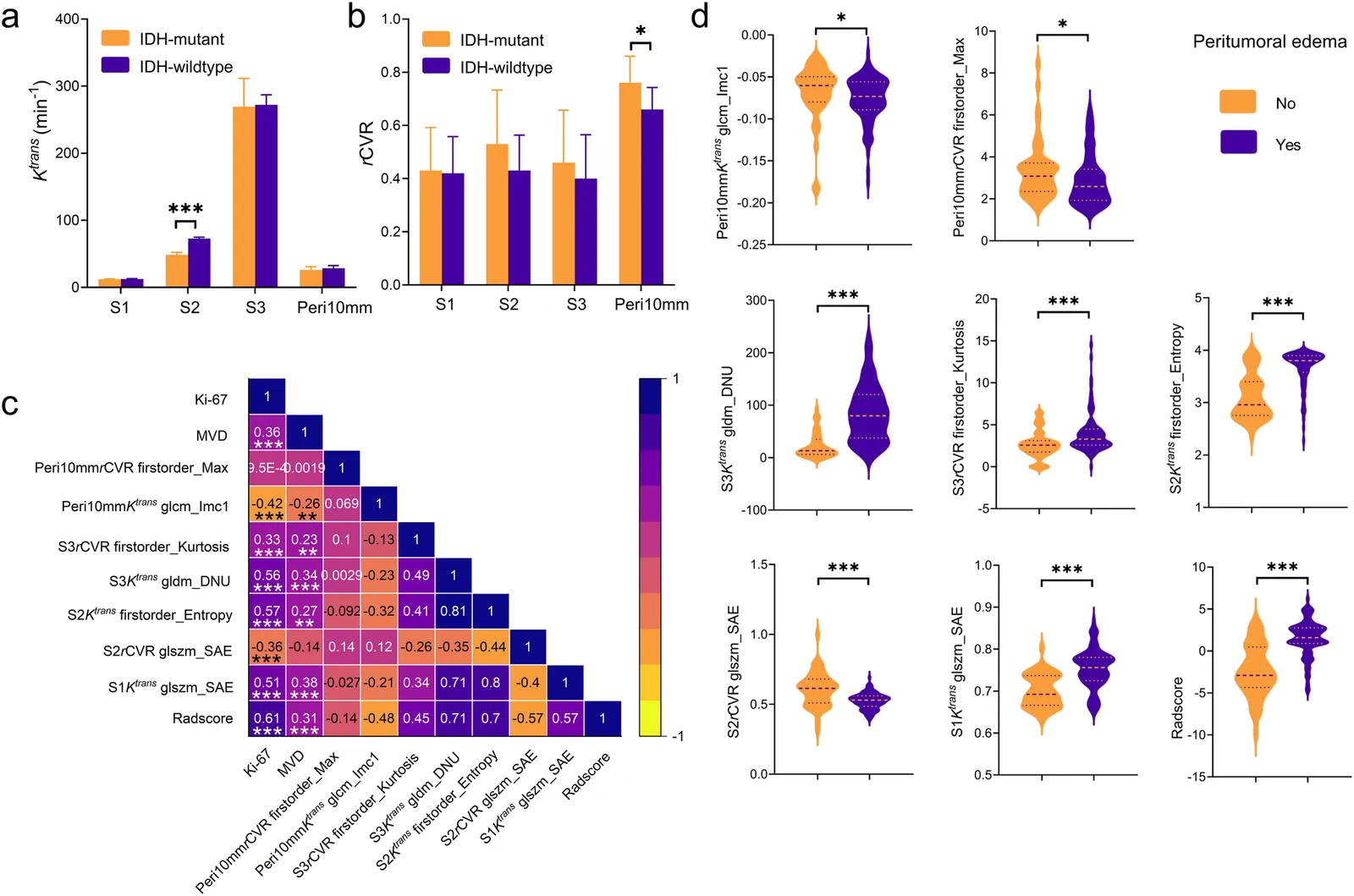
**Associations of imaging-derived features with IDH status, histopathological markers, and peritumoral edema.** (a-b) Comparison of DCE-derived *K*^trans^ and BOLD-derived *r*CVR across IDH groups in the intratumoral subregions and Peri10mm compartment. (c) Correlation matrix of key radiomic features (including Radscore) with Ki-67 proliferation index and MVD. (d) Comparison of key radiomic features between edema-positive and edema-negative tumors. *Note*: The values in the heatmaps are Spearman correlation coefficients (r). The color bar indicates the strength of the correlation (r value). ∗, p < 0.05; ∗∗, p < 0.01; ∗∗∗, p < 0.001. *Abbreviations*: DCE-MRI, dynamic contrast-enhanced magnetic resonance imaging; rs-BOLD-fMRI, resting-state blood oxygen level-dependent functional magnetic resonance imaging; *K*^trans^, volume transfer constant; *r*CVR, relative cerebrovascular reactivity; S1, subregion1; S2, subregion2; S3, subregion3; Peri10mm, peritumoral 10 mm region; IDH, isocitrate dehydrogenase; MVD, microvessel density; Radscore, radiomic score; glcm, gray-level co-occurrence matrix; Imc1, information measure of correlation1; glrlm, gray-level run length matrix; glszm, gray-level size zone matrix; Max, maximum; RLN, run-length non-uniformity; SAE, small area emphasis; DNU, dependence non-uniformity; SAHGLE, small area high gray level emphasis.

#### Exploratory associations with histopathological and edema-related measures

Selected subregion-derived radiomic features showed varying patient-level correlations with Ki-67 and MVD (Fig. 5c and Supplementary Fig. S4a–b). The integrated Radscore showed the strongest reported correlation with Ki-67 (r = 0.61, p < 0.001). *K*^trans^ first-order Entropy derived from Subregion 2 correlated with Ki-67 (r = 0.57) and MVD (r = 0.27). Selected imaging features were also compared between patients with and without peritumoral edema (Fig. 5d and Supplementary Table S8). The Subregion 3 shape feature LeastAxisLength differed between the two groups (z = −6.960, p < 0.001; Supplementary Table S8).

### Exploratory evaluation of the ITH score

#### Quantification of intratumoral heterogeneity

The first principal component (PC1) explained 62.89% of the variance and was used to derive the ITH score (Supplementary Fig. S5a). Variable loading analysis further revealed the contribution of each input feature to PC1 (Supplementary Fig. S5b). The ITH score calculated based on PC1 demonstrated a significant difference between IDH-mutant and wildtype groups (0.40 vs. 0.79, p < 0.001; Fig. 6a, Supplementary Table S6) and showed good discriminative ability for IDH genotyping (AUC = 0.824; Fig. 6b).

**Fig. 6:**
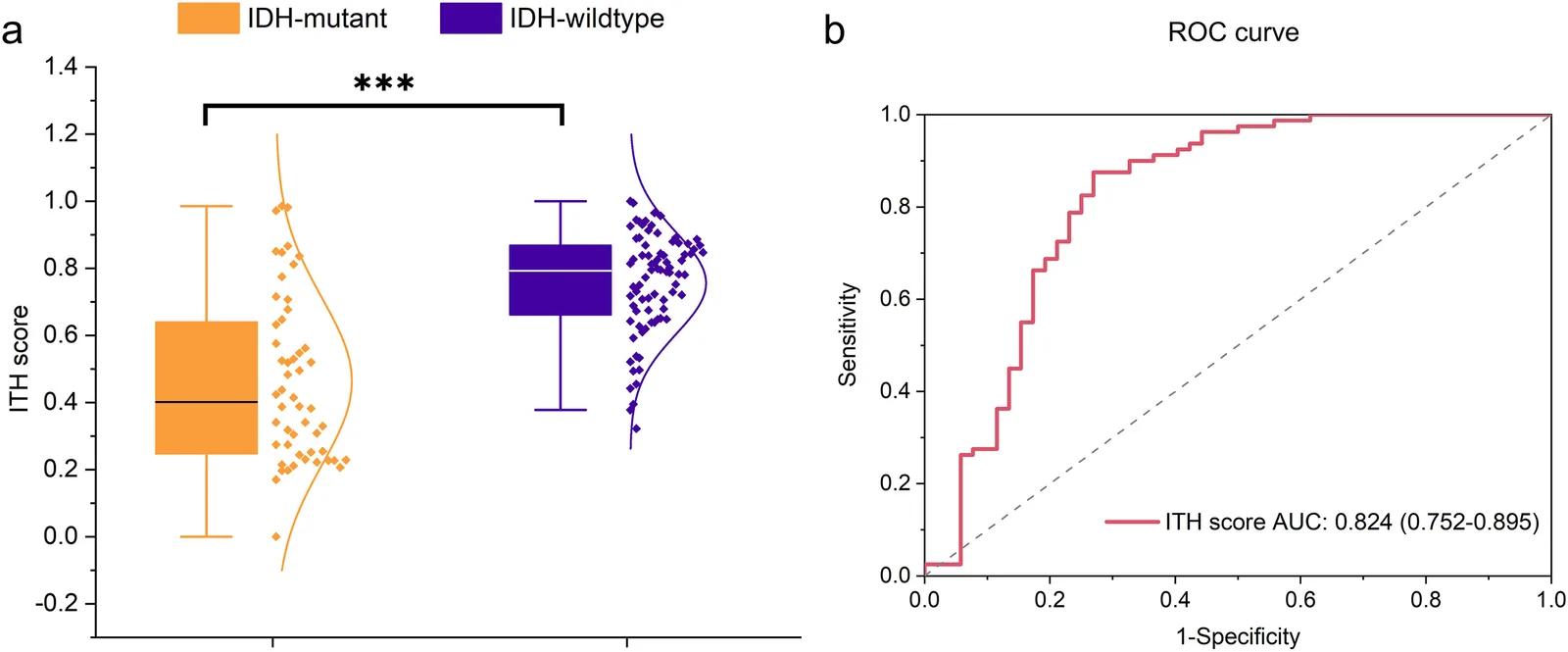
**Association of the ITH score with IDH status and its discrimination performance.** (a) Distribution of ITH scores in IDH-mutant vs. wildtype gliomas (box-violin plot). (b) ROC curve for IDH status prediction. *Abbreviations*: ITH, intratumoral heterogeneity; IDH, isocitrate dehydrogenase; ROC, receiver operating characteristic; AUC, area under the curve.

Additionally, we conducted an exploratory subgroup analysis to investigate the potential association between the ITH score and EGFR amplification status. Among the 50 patients with available EGFR testing results (19 IDH-mutant and 31 IDH-wildtype), no significant differences in ITH score were observed between EGFR-amplified and non-amplified subgroups within the same IDH genotype category (all p > 0.05; Supplementary Table S9). However, further analysis revealed that regardless of EGFR amplification status, IDH-wildtype patients consistently showed significantly higher ITH scores compared to IDH-mutant patients (EGFR-amplified subgroup: 0.76 vs. 0.29, p < 0.001; EGFR non-amplified subgroup: 0.76 vs. 0.45, p = 0.001).

#### Exploratory analysis of progression-free survival

Exploratory PFS analyses based on the available follow-up data are presented in Supplementary Results 2, Supplementary Fig. S6a–b, and Supplementary Table S10–S12.

#### Exploratory correlations with HIF-1α and VEGF-A expression

In the exploratory subset of 20 patients with IDH-wildtype gliomas, the correlations of the ITH score with HIF-1α expression (r = 0.39, p = 0.09; Supplementary Fig. S6c) and VEGF-A expression (r = 0.20, p = 0.41; Supplementary Fig. S6d) were not statistically significant.

## Discussion

This study evaluated a multiscale habitat imaging framework integrating DCE-MRI-derived *K*^trans^ and rs-BOLD-fMRI-derived *r*CVR to characterize intratumoral and peritumoral functional heterogeneity and explore noninvasive IDH genotyping. In the internal temporal test cohort, the Habitat and Habitat-Peri10mm models yielded AUC point estimates of 0.854 and 0.914, respectively, compared with 0.753 for the Whole-tumor model. However, paired DeLong tests did not demonstrate statistically significant differences between the Habitat-Peri10mm model and either the Habitat model (uncorrected p = 0.173) or the Whole-tumor model (uncorrected p = 0.054). These findings support further evaluation of combined intratumoral and peritumoral habitat information but do not establish superior discrimination. Beyond model performance, the framework characterizes regional variation in vascular permeability and functional reserve across three intratumoral subregions and the anatomically defined peritumoral compartment. Associations of selected imaging features with Ki-67, MVD, and peritumoral edema suggest potential biological relevance and provide a basis for exploring how these regional imaging patterns relate to glioma pathophysiology.

### Physiological rationale for DCE-BOLD coupling

Habitat-based glioma characterization has been investigated using structural MRI [36], perfusion imaging [24], and multiparametric combinations [28], [37]. A perfusion–diffusion habitat study reported an AUC of 0.815 for IDH classification [27], while a diffusion-derived vessel-density habitat model incorporating clinical variables reported an AUC of 0.979 [30]. Metabolic–hemodynamic habitat imaging has also been explored in relation to PFS [29]. These studies establish an important foundation for the present work. Differences in patient populations, predictor sets, and validation designs limit direct comparison of their reported AUCs with our internal test estimate.

The specific contribution of the present framework is the joint characterization of permeability-related and reactivity-related imaging variation. Whereas the *K*^trans^–ADC approach combines DCE-derived contrast transfer with diffusion-sensitive tissue information [28], the *K*^trans^–*r*CVR pairing incorporates an estimate of vascular functional reserve derived from resting-state BOLD imaging [38], [39]. This enables assessment of whether regions with relatively elevated *K*^trans^ retain or exhibit reduced *r*CVR, providing a physiological basis for describing permeability–reactivity coupling patterns. The potential value of this pairing lies in the complementary information it captures; its comparative diagnostic benefit requires evaluation within the same cohorts and validation framework.

Extending the analysis to the anatomically defined Peri10mm compartment allows intratumoral patterns to be considered alongside imaging variation in the surrounding tissue [31], [32], [40]. The Peri10mm model yielded an internal test AUC point estimate of 0.776, suggesting that this compartment may contain information associated with IDH status. The present analysis does not establish its independent predictive value or demonstrate incremental discrimination. The combined intratumoral–peritumoral framework therefore warrants further evaluation in independent cohorts.

### Biological interpretation of imaging-defined vascular patterns

The relative *K*^trans^–*r*CVR profiles provide a framework for interpreting regional variation in vascular permeability and functional reserve. Their biological interpretation must also consider the cohort composition: all IDH-wildtype tumors were WHO grade 4, whereas IDH-mutant tumors spanned grades 2–4. The imaging patterns may therefore reflect a combination of IDH-associated, grade-related, and tumor-type-related characteristics. Considered alongside the observed histopathological and edema associations, these profiles suggest possible biological processes underlying the regional patterns described below.

Subregion 1 showed a relatively low-permeability/low-reserve profile (Table 3), while a *K*^trans^-derived texture feature correlated positively with Ki-67 and MVD. These observations may reflect different aspects of regional heterogeneity: mean parameter values summarize the overall imaging profile, whereas texture features characterize spatial variation within the subregion. Spatial transcriptomic studies of glioblastoma have identified different molecular and cellular profiles in pseudopalisading regions around necrosis and areas of microvascular proliferation [41]. Against this background, one possible explanation for our findings is heterogeneous tissue composition at interfaces between necrotic and viable tumor, where low average values may coexist with localized variation associated with proliferative and vascular features. This interpretation warrants evaluation using spatially matched imaging and histopathology.

Subregion 2 showed a moderate-permeability/high-reserve profile. Its *K*^trans^ first-order Entropy feature ranked highest in the SHAP analysis and correlated with Ki-67 (r = 0.57) and MVD (r = 0.27), while an *r*CVR texture feature from the same subregion had the most negative coefficient in the Radscore model. These findings suggest that imaging heterogeneity within Subregion 2 may be associated with proliferative activity and vascular characteristics. Experimental work has shown that glioma stem cells can induce different endothelial responses, including angiogenesis and vascular enlargement, through tumor–endothelial interactions [42]. This provides a biological context for investigating how regional permeability–reactivity patterns relate to tumor activity and vascular remodeling.

Subregion 3 showed a high-permeability/low-reserve profile, consistent with the imaging-defined vascular uncoupling pattern proposed in this study. The coexistence of relatively elevated *K*^trans^ and reduced *r*CVR suggests that increased vascular permeability may occur alongside impaired vascular reserve. Previous physiological MRI studies have demonstrated regional heterogeneity in neovascularization and oxygen metabolism in glioblastoma [2], providing a broader context for this functional mismatch. In our cohort, the Subregion 3 shape feature LeastAxisLength differed between tumors with and without peritumoral edema (z = −6.960, p < 0.001; Supplementary Table S8). Experimental studies have also shown that interactions between glioblastoma cells and perivascular stromal cells can influence tumor invasion and vascular organization [40], [43]. Together, these observations motivate further investigation of the relationship between the spatial morphology of this vascular uncoupling pattern and the surrounding tumor microenvironment.

The anatomically defined Peri10mm compartment showed a relatively low-permeability/high-reserve profile. Within this compartment, *r*CVR was lower in IDH-wildtype than in IDH-mutant tumors (p < 0.05; Fig. 5b and Supplementary Table S6), and selected *K*^trans^-derived texture features showed negative correlations with Ki-67 and MVD. These observations suggest that the peritumoral imaging profile may capture aspects of the surrounding tissue response associated with tumor characteristics. Previous research has reported an association between IDH status and the edema-to-tumor ratio [31], while studies of peritumoral tissue have described a heterogeneous environment containing infiltrating tumor cells, reactive glial cells, and vascular components [32]. This tissue complexity provides a biological context for interpreting the Peri10mm profile and supports further investigation of vascular changes beyond the tumor margin.

Together, the three intratumoral subregions and the anatomically defined peritumoral compartment characterize regional variation in permeability–reactivity coupling within and around the tumor. Their imaging profiles and associations with histopathological and edema-related measures provide a biologically informed framework for investigating glioma heterogeneity. This regional characterization offers a basis for future studies integrating habitat imaging with spatially matched tissue sampling to clarify the relationships between vascular function and tumor biology.

### ITH score and IDH-associated imaging heterogeneity

The ITH score differed between IDH-mutant and IDH-wildtype gliomas and provides a quantitative summary of the spatial composition of the imaging-defined intratumoral subregions. The association of the ITH score with peritumoral edema suggests a relationship between intratumoral imaging heterogeneity and the surrounding tissue response. Previous studies have reported IDH-associated vascular gene expression patterns and investigated MRI-derived features for predicting IDH status and VEGF expression [44], [45]. These findings provide a biological context for further investigation of the observed IDH-associated differences in the ITH score within the broader molecular landscape of glioma [46]. Larger cohorts integrating imaging with spatially matched tissue assessment are needed to clarify the biological processes underlying this imaging phenotype.

### Limitations

This study has several limitations. First, the single-center cohort included 92 training and 40 internal test patients, leaving a risk of overfitting despite staged feature selection. The original five-fold cross-validation followed training-cohort preprocessing and feature selection and may have produced optimistic estimates; the separate internal test AUC of 0.914 also requires external validation. Second, all 80 IDH-wildtype tumors were grade 4 glioblastomas, whereas the 52 IDH-mutant tumors spanned grades 2–4, with only six grade 4 cases. The observed imaging differences and classification performance may therefore reflect grade and tumor type as well as IDH status. Within-IDH-mutant grade comparisons do not separate these effects. Third, the low silhouette score (0.115) indicates overlapping intratumoral imaging profiles. Relatively stable bootstrap core assignments do not establish clearly separated biological compartments or reproducibility in other cohorts. Fourth, histopathological measurements were obtained at the patient level without spatial matching. The correlations of the ITH score with HIF-1α and VEGF-A expression were not statistically significant in the 20-patient subset, leaving its relationship with hypoxia-related biology unresolved. Finally, follow-up was available for 67 of 132 patients, with 29 progression events, limiting the precision and representativeness of the exploratory survival findings reported in the Supplementary Materials. Larger independent cohorts with spatially matched tissue assessment and more complete follow-up are needed.

## Conclusion

In conclusion, this study presents a DCE-BOLD-based multiscale habitat imaging framework that noninvasively characterizes intratumoral and peritumoral functional heterogeneity in glioma. By integrating *K*^trans^ and *r*CVR, the framework captures spatial variation in permeability–reactivity coupling across three GMM-defined intratumoral subregions and an anatomically defined peritumoral compartment, and yields a high internal test AUC point estimate for IDH genotyping. The ITH score further characterizes imaging heterogeneity associated with IDH status. Collectively, these findings provide a biologically informed framework for linking imaging-defined vascular patterns to tumor pathophysiology. Future prospective multicenter studies with external validation and imaging–histopathology coregistration are needed to confirm these findings and advance clinical translation.

## Author contributions

D.D.S contributed significantly to the conceptualization, methodology, formal analysis, investigation, data curation, and writing—original draft.

Y.T.W contributed to formal analysis and investigation.

B.R.L, Y.L, P.X.G, and Y.Q.M contributed to data curation.

H.Y.Q contributed to formal analysis and provided support in the statistical methods.

S.R.N contributed to formal analysis.

Y.L.J provided support in MRI techniques, data processing and analysis.

Y.S provided support in data processing and analysis.

G.G.F contributed to resources.

Z.X.L contributed to formal analysis, investigation and writing—review.

M.C contributed significantly to the conceptualization, methodology, formal analysis, investigation, resources, funding acquisition, and writing—review & editing.

All the authors contributed to the manuscript.

## Declarations of interest statement

The authors declare no potential conflicts of interest with respect to the research, authorship, and publication of this article.
