# Supplementary material for: Multiscale DCE-BOLD MRI reveals vascular uncoupling patterns for IDH genotyping in adult glioma: A prospective habitat-based study
Source: Neurotherapeutics. 2026 Oct 30;24(1):e01114. doi: 10.1016/j.neurot.2026.e01114 (PMC13743516; doi:10.1016/j.neurot.2026.e01114)
Supplement: Supplementary file 1 [file mmc1.docx]

**SUPPLEMENTARY MATERIALS**

**Supplementary Methods 1 – Detailed rs-BOLD-fMRI Preprocessing, *r*CVR Calculation and DCE-MRI Pharmacokinetic Modeling**

Resting-state BOLD-fMRI data were acquired using a gradient-echo echo-planar imaging (ep2d_bold) sequence. To achieve high spatial resolution, parameters were set as follows: repetition time (TR) = 2000 ms, echo time (TE) = 30 ms, voxel size = 2.0 × 2.0 × 2.0 mm³ (isotropic), field of view (FOV) = 240 × 240 mm², matrix = 120 × 120, number of slices = 54 (acquired contiguously in the axial plane to ensure coverage of the cerebral hemispheres). A total of 210 time points were acquired. During scanning, participants were instructed to stay awake, remain relaxed, fixate on a central crosshair, and keep their head as still as possible.

DCE‑MRI was performed using a 3D volumetric interpolated breath‑hold examination sequence based on the Golden‑angle Radial Sparse Parallel (GRASP) technique. This sequence integrates golden‑angle radial *k*‑space sampling, parallel imaging (using the Generalized Autocalibrating Partially Parallel Acquisitions, GRAPPA, technique), and compressed sensing reconstruction, enabling high spatiotemporal resolution dynamic acquisition during free breathing. Prior to the dynamic series, the following calibration and quantitative scans were performed sequentially: (1) A B1 mapping sequence (TR = 4350 ms, TE = 1.97 ms, FOV = 380 × 380 mm², bandwidth = 490 Hz/Px) was acquired for subsequent correction of signal bias induced by radiofrequency field inhomogeneity. (2) Pre‑contrast T1 mapping was obtained using a dual‑flip‑angle (2° and 10°) 3D volumetric interpolated breath‑hold examination (VIBE) sequence (TR = 5.1 ms, TE = 2.02 ms, bandwidth = 260 Hz/Px) to provide baseline T1 values for quantitative contrast agent concentration calculation in the subsequent pharmacokinetic modeling. (3) The dynamic enhancement scan commenced with the acquisition of 3 baseline volumes as a pre‑contrast reference. Subsequently, gadobutrol (Gadovist; Bayer Schering Pharma, Berlin, Germany) was administered intravenously via an antecubital vein using a dual‑syringe power injector at a flow rate of 2.0 mL/s (dose: 0.1 mmol/kg body weight), followed immediately by a 20 mL saline flush at the same rate. Dynamic image acquisition was synchronized precisely with the contrast agent injection and continued from the start of the bolus, yielding a total of 39 dynamic volumes. The core acquisition parameters for the GRASP sequence were as follows: TR = 4.2 ms, TE = 1.96 ms, flip angle = 12°, slice thickness = 0.9 mm, no gap, number of slices = 72, bandwidth = 450 Hz/Px, FOV = 450 × 450 mm², reconstruction matrix = 230 × 230 (resulting in an approximately isotropic voxel size of 0.9 × 0.9 × 0.9 mm³). The number of radial views was 1397, providing a temporal resolution of 10.6 seconds per dynamic volume.

First, *rs*-BOLD-fMRI data processing was conducted using Statistical Parametric Mapping (SPM12, University College London) and inhouse Matlab (R2023a, MathWorks, Natick, MA) scripts. Following a previously proposed pipeline for resting-state CVR mapping[1], the BOLD-fMRI data first underwent motion correction, spatial smoothing (FWHM = 8 mm), linear detrending and temporal filtering with a frequency range of [0, 0.1164 Hz]. Then, reference signal time course was extracted from healthy brain regions unaffected by the glioma. Finally, the *r*CVR map was calculated by a general linear regression model between the voxel-wise BOLD time course and the reference signal, and subsequently normalized by whole-brain average value.

Then, quantitative post‑processing of DCE‑MRI data was performed on a Siemens syngo.via workstation using the MR 4D Tissue module. The processing was based on the DCE series and its corresponding calibration sequences, with the following steps: (1) Data Reconstruction: Compressed sensing‑based iterative reconstruction was applied to the raw GRASP sequence data to generate a 4D dynamic series with a temporal resolution of 10.6 s and 39 time points. Pre‑contrast 3D VIBE T1 mapping data acquired with dual flip angles (2° and 10°) were also exported. (2) Motion Correction: The eighth pre‑contrast time point was used as a reference to rigidly align all subsequent time points, correcting for head motion during scanning. (3) Cross‑Sequence Registration: The T1 mapping images were spatially aligned with the motion‑corrected dynamic reference time point to ensure anatomical consistency. (4) Pharmacokinetic Modeling: Baseline T1 values derived from the dual‑flip‑angle sequences were used to convert the dynamic signal intensity curves into contrast‑agent concentration‑time curves for each voxel. A patient-specific arterial input function (AIF) was manually defined by placing a region of interest (~3 voxels) within the lumen of the ipsilateral internal carotid artery. Using the standard Toft’s model[2] and nonlinear least‑squares fitting, quantitative parameter maps—including *K*^trans^, *V_e_*, and *K_ep_* (*K_ep_* = *K*^trans^/*V_e_*)—were generated voxel‑wise.

**Supplementary Methods 2 – Spatial Registration and Normalization Workflow**

For subsequent multiparametric analysis, the *r*CVR, *K*^trans^ maps, and conventional sequences (T1CE, T2‑FLAIR) underwent systematic preprocessing. The mean BOLD image (already registered to standard space) and the 39th time point of the dynamic series (showing peak enhancement) were selected as anatomical references. Using the MPQuan module in MR Station software (Chengdu Zhongying Medical Technology Co., Ltd.), the following steps were applied uniformly: (1) Intensity Normalization: Histogram matching was used to standardize signal intensities across all images to a common range of 0-1700, reducing inter‑scan variability. (2) Isotropic Resampling: All images were resampled to a uniform in‑plane resolution of 0.8 × 0.8 mm².

Spatial normalization was then performed to achieve precise anatomical alignment. Preprocessed T1CE and T2‑FLAIR images were separately rigidly registered (six degrees of freedom) to the mean BOLD image and the dynamic reference time point, respectively. The resulting transformation matrices were applied synchronously to the corresponding *r*CVR and *K*^trans^ maps. This workflow ensured that all multiparametric images were consistently aligned within the same anatomical coordinate system, preserving quantitative information while providing a reliable spatial foundation for voxel‑wise comparison and integrated analysis.

**Supplementary Methods 3 – Quality Control Protocol for VOI Delineation**

To mitigate potential errors introduced by manual volumes of interest (VOIs) delineation, a rigorous multi-tier quality control protocol was implemented, beginning with interobserver consistency validation where primary contouring performed by a board-certified neuroradiologist (5 years of experience) was independently validated by a senior neuroradiologist (10+ years of experience) under blinded conditions, quantified through intraclass correlation coefficients (ICC > 0.85 for volumetric overlap) and Cohen’s κ coefficient (κ > 0.75 for categorical spatial congruence). Additionally, intraobserver reproducibility was systematically assessed by having the primary annotator repeat delineation on a randomly selected subset of 20% of cases after a 2-week interval, with intraclass correlation coefficients (ICC > 0.90) serving as the threshold for confirming intraobserver consistency. This dual-layered approach ensured both spatial reliability across observers and temporal stability within the same annotator.

**Supplementary Methods 4 – Radiomics Analysis**

**Radiomic Feature Extraction and Processing**

Image feature extraction was performed using the open-source FeAture Explorer (FAE, Version 0.6.0; https://github.com/salan668/FAE) platform incorporating PyRadiomics (Version 3.0)[3]. From each quantitative parametric map of DCE (*K*^trans^) and BOLD (*r*CVR), we systematically extracted 107 Imaging Biomarker Standardization Initiative (IBSI)-compliant features, encompassing 14 morphological, 18 first-order statistical, and 75 textural features. Because the two parametric maps shared the same regional segmentation mask, only one set of 14 shape features was retained for each VOI. Each region therefore contributed 200 candidate features: 14 shape features and 93 first-order and texture features from each parametric map. Separate feature sets were constructed for the whole-tumor VOI, each of the three intratumoral subregions, and the Peri10mm VOI, with feature selection subsequently performed within each regional feature set. For texture feature calculation, image intensities were discretized using a fixed bin width of 25, consistent with IBSI guidelines. The derived radiomic features underwent temporal stratification to partition the dataset into temporally training (70%) and independent internal test (30%) cohorts, preserving the chronological sequence of patient enrollment while preventing temporal data leakage between development and validation phases.

**Feature Selection and Model Construction**

Model development was performed using the Model Exploration module of FeAture Explorer (FAE v0.6.0). Radiomic features were normalized using mean normalization to the range of −0.5 to 0.5. Redundant features were removed using Pearson correlation filtering with a threshold of 0.99. Feature selection was performed using recursive feature elimination (RFE), and classification was performed using a support vector machine (SVM). Five-fold cross-validation was subsequently used to evaluate classifier performance on the selected feature sets within the training cohort. The preceding preprocessing and feature-selection steps were not repeated within individual cross-validation folds. The internal test cohort was excluded from preprocessing parameter estimation, feature selection, hyperparameter tuning, and model selection. For internal test evaluation, the preprocessing transformations and selected feature set determined from the training cohort were applied unchanged, and predictions were generated using the classifier fitted on the training cohort.

**Additional Internal Sensitivity Analyses**

Additional internal sensitivity analyses comprised leave-one-out cross-validation (LOOCV) of the evaluated radiomic models and a permutation test. These analyses were restricted to the training cohort of 92 patients and used the extracted regional radiomic feature sets, with the existing GMM-defined partitions held fixed. The internal test cohort was not used in these analyses.

In each outer LOOCV iteration, one patient was held out and model development was performed using the remaining 91 patients. Normalization, correlation-based redundancy reduction, regional feature selection, feature integration where applicable, and subsequent classifier fitting were repeated using the training data. Feature-count selection and classifier hyperparameter tuning were conducted using inner five-fold cross-validation, with preprocessing and feature selection fitted separately within each inner training fold. The selected pipeline was then refitted on the 91 patients and applied to the held-out patient. Predictions from all 92 outer iterations were pooled to calculate the AUC.

The 95% confidence intervals for the LOOCV AUCs were estimated using 2,000 stratified bootstrap resamples of the paired patient-level IDH labels and held-out prediction scores. Patients were sampled with replacement within each IDH group, preserving the original group sizes. The AUC was calculated for each resample, and the 2.5th and 97.5th percentiles of the bootstrap distribution defined the confidence interval.

For the permutation test, IDH labels were randomly permuted among patients in the training cohort 1,000 times. The observed statistic was the AUC calculated from pooled held-out predictions obtained using outer five-fold cross-validation with the original labels. For each permutation, the same evaluation procedure was repeated, including preprocessing, regional and combined feature selection, inner five-fold model selection, and classifier fitting within the appropriate training folds. The same cross-validation splitting rules and prespecified random seeds were used for the original and permuted labels. The permutation p value was calculated as (b + 1)/(1,000 + 1), where b was the number of permuted AUCs greater than or equal to the observed AUC.

**Supplementary Methods 5 – Histopathological Assessment (Ki‑67, MVD, HIF‑1α, VEGF‑A, EGFR)**

High-risk molecular markers associated with adult diffuse gliomas—including isocitrate dehydrogenase (IDH) mutations, 1p/19q codeletion, O6-methylguanine-DNA methyltransferase (MGMT) promoter methylation, telomerase reverse transcriptase (TERT) promoter mutations, cyclin-dependent kinase inhibitor 2A/B (CDKN2A/B) homozygous deletions, and epidermal growth factor receptor (EGFR) amplification—were systematically characterized using standardized molecular pathology workflows. IDH1/2 mutations at hotspot codons R132 and R172 were detected in resected specimens through amplification-based methods (polymerase chain reaction, PCR), with pathogenic variants at either locus classified as IDH mutations. TERT promoter mutations (C228T/C250T) were identified via PCR amplification, where variants at either site were defined as TERT mutations. MGMT promoter methylation status was determined by methylation-specific PCR. 1p/19q codeletion status was assessed by fluorescence in situ hybridization (FISH), with a 1p/19q probe signal ratio < 0.70 defining codeletion. FISH assays concurrently evaluated CDKN2A/B homozygous deletions (probe signal loss in > 30% tumor cells) and EGFR amplification (gene-to-centromere ratio ≥ 2.0). The Ki-67 proliferation index and MVD were quantified by immunohistochemical staining. The Ki-67 proliferation index was expressed as the percentage of positively stained tumor nuclei. Five hot spots were selected under high-power magnification ( × 400), with at least 1000 tumor cells counted per field. The final proliferation index was determined as the mean percentage of positive nuclei across the five fields. MVD was assessed on CD34-stained sections according to the Weidner method. All evaluations were performed independently by two pathologists, and discrepancies exceeding 5% were resolved by consensus.

Formalin-fixed paraffin-embedded (FFPE) tissue samples were collected from 20 patients with IDH-wildtype GBM. Consecutive sections of 4 μm thickness were prepared for immunohistochemical detection of HIF-1α and VEGF-A expression. The staining procedure was as follows: sections were baked at 60°C for 2 h, dewaxed in xylene, and rehydrated through a graded ethanol series. Antigen retrieval was performed using different methods: high-pressure heating in retrieval solution for 10 min for HIF-1α, and microwave heating in EDTA retrieval solution (low power, 10 min) for VEGF-A. After retrieval, sections were incubated with 3% H₂O₂ for 15 min at room temperature to block endogenous peroxidase activity, followed by blocking with normal goat serum for 15 min at room temperature. Primary antibodies were applied overnight at 4°C in a humidified chamber: anti-HIF-1α (Proteintech, 20900-1-AP, 1:200 dilution) and anti-VEGF-A (Proteintech, 66828-1-IG, 1:1000 dilution). The next day, sections were incubated with corresponding HRP-conjugated secondary antibodies for 30 min at 37°C: goat anti-rabbit IgG (Jackson, 164117, 1:200 dilution) for HIF-1α, and goat anti-mouse IgG (Jackson, 164315, 1:200 dilution) for VEGF-A. Immunoreactivity was visualized using DAB chromogen, with reaction time monitored microscopically and terminated by washing with water upon appearance of brownish-yellow positive signals. Sections were counterstained with hematoxylin, dehydrated through graded ethanol, cleared in xylene, and mounted with neutral balsam.

Image acquisition and quantitative analysis were performed as follows: five non-overlapping fields (avoiding necrotic and hemorrhagic areas) were randomly captured per section under a light microscope at ×400 magnification with consistent exposure parameters. Images were imported into FiJi software (2.14.0/Java 8, https://imagej.org) for quantitative analysis under the guidance of two senior pathologists. Color deconvolution algorithm was applied to separate DAB-positive signals from hematoxylin counterstaining. Based on subcellular localization characteristics, HIF-1α positivity was defined as brownish-yellow granules in the nucleus, and the percentage of DAB-positive nuclear area was calculated after nuclear segmentation. VEGF-A positivity was defined as brownish-yellow staining in the cytoplasm and/or cell membrane, and the percentage of DAB-positive area was measured within manually delineated tumor parenchyma. The mean value from five fields per section was calculated as the final expression level for each marker.

**Supplementary Methods 6 – ITH Score Calculation**

Although the number of GMM clusters was uniformly predefined, the actual number of subregions generated varied according to the inherent heterogeneity of each tumor. The calculation procedure consisted of the following steps: (1) Feature Extraction: For each patient, the spatial volume proportion of each actually generated subregion was extracted. Shannon entropy was then computed based on information theory [Shannon entropy = -∑(p_i × log₂(p_i))] to quantify the disorder in spatial composition[4]. These metrics collectively characterized tumor heterogeneity; (2) Matrix Construction and Standardization: A feature matrix was constructed for each patient, containing the volume proportions of all subregions, actual number of subregions, and Shannon entropy value (five features in total). All features were subsequently standardized; (3) Principal Component Analysis (PCA) and Score Generation: PCA was performed on the standardized feature matrix. The first principal component (PC1) was extracted as the initial ITH score. The PC1 values were then linearly rescaled to a range of [0-1] to obtain the final standardized ITH score, where higher values indicate greater spatial heterogeneity. This workflow ensured the compatibility and comparability of heterogeneity patterns across all patients. All computations were carried out using R (v4.5.2).

**Supplementary Methods 7 – Exploratory PFS Analysis**

The prognostic value of the ITH score was assessed using PFS (defined according to the Response Assessment in Neuro-Oncology [RANO] criteria[5]) as the primary endpoint. First, patients were stratified into high- and low-risk groups based on the median ITH score. Survival differences were compared using Kaplan-Meier curves and the log-rank test. Subsequently, the discriminative ability of the ITH score-based model was quantified by the concordance index (C-index) with 1000 bootstrap resamples. Finally, univariate and multivariate Cox regression analyses evaluated its association with PFS, with the latter adjusted for age and edema. These analyses were exploratory in nature to determine potential associations.

**Supplementary Results**

**Supplementary Results 1 – GMM Clustering and Bootstrap Stability Analysis**

Bootstrap analysis was performed to assess the stability of voxel-wise subregion assignments within the training cohort using 100 resamples with replacement. For each resample, a three-component GMM was refitted, and the frequency with which each voxel was assigned to the same subregion as in the original clustering was calculated. Voxels with assignment frequencies greater than 0.7 were designated as “stable cores”, whereas lower frequencies indicated greater assignment uncertainty. More than 70% of voxels met the stable-core criterion. These results indicated relatively stable core assignments alongside substantial overlap in the joint *K*^trans^–*r*CVR feature space (silhouette score, 0.115).

**Supplementary Results 2 – Exploratory Survival Analysis (limited by follow-up)**

Follow-up data were available for 67 of 132 patients (51%), with 29 disease progression events. Due to the high loss to follow-up (49%), the following survival analyses are strictly exploratory and presented only to generate hypotheses; no definitive prognostic claims are made.

Using the median ITH score (0.71) to stratify patients, the high-ITH group showed a nominally shorter PFS than the low-ITH group (median 8.2 months vs. not reached; log-rank p = 0.015) **(Supplementary Fig. S6a)**. In a multivariable Cox model adjusted for age and edema, the ITH score remained associated with PFS (HR = 13.30; 95% CI: 1.29–137.19; p = 0.03), but the extremely wide confidence interval reflects the small number of events (n = 29) and the high loss to follow-up **(Supplementary Fig. S6b, Supplementary Tables S10–S12)**. The C-index (based on limited data) was 0.611 (95% CI: 0.491–0.712). These exploratory results should not be interpreted as evidence of independent prognostic value.

**Supplementary Results 3 – Additional Internal Sensitivity Analyses**

The Habitat-Peri10mm model yielded a LOOCV AUC of 0.858 (95% CI, 0.768–0.948) in the training cohort. LOOCV results for the evaluated radiomic models are presented in Supplementary Table S13. The Habitat-Peri10mm model’s cross-validated AUC was higher than expected under randomly permuted IDH labels (1,000 permutations; p < 0.001).

**References:**

1. Liu P, Liu G, Pinho MC, Lin Z, Thomas BP, Rundle M, et al. Cerebrovascular Reactivity Mapping Using Resting-State BOLD Functional MRI in Healthy Adults and Patients with Moyamoya Disease. Radiology 2021;299(2):419-425.

2. Van Elburg JM, Korobova NV, Islam MM, Troelstra MA, Gurney-Champion OJ. Uncertainty estimates in pharmacokinetic modelling of DCE-MRI. Med Image Anal 2026;109:103881.

3. Song Y, Zhang J, Zhang Y, Hou Y, Yan X, Wang Y, et al. FeAture Explorer (FAE): A tool for developing and comparing radiomics models. PLoS One 2020;15:e237587.

4. Shi Z, Huang X, Cheng Z, Xu Z, Lin H, Liu C, et al. MRI-based Quantification of Intratumoral Heterogeneity for Predicting Treatment Response to Neoadjuvant Chemotherapy in Breast Cancer. Radiology 2023;308(1):e222830.

5. Wen PY, van den Bent M, Youssef G, Cloughesy TF, Ellingson BM, Weller M, et al. RANO 2.0: Update to the Response Assessment in Neuro-Oncology Criteria for High- and Low-Grade Gliomas in Adults. J Clin Oncol 2023;41:5187-5199.

| **MR sequences** | **T2WI** | **T2-FLAIR** | **T1CE** | **DCE-MRI** | | **rs-Bold-fMRI** | |
| --- | --- | --- | --- | --- | --- | --- | --- |
|  |  |  |  | **Vida** | **Prisma** | **Vida** | **Prisma** |
| TR (ms) | 4200 | 8300 | 1800 | 4.20 | 4.20 | 2000 | 2000 |
| TE (ms) | 97 | 99 | 31 | 1.96 | 1.96 | 30 | 30 |
| FA (degree) | 120 | 110 | 120 | 12 | 12 | 80 | 80 |
| Bandwidth (Hz/pixel) | 280 | 279 | 386 | 450 | 450 | 1984 | 1984 |
| FOV (mm) | 230 | 230 | 230 | 450 | 450 | 240 | 240 |
| Matrix | 416 | 320 | 288 | 230 | 230 | 120 | 120 |
| Thickness (mm) | 5 | 5 | 5 | 0.9 | 0.9 | 2 | 2 |
| Acquisition time (s) | 34 | 100 | 126 | 438 | 438 | 433 | 433 |

**Table S1. MRI acquisition parameters**

*Abbreviations*: MRI, magnetic resonance imaging; T2WI, T2-weighted imaging; T2-FLAIR, T2-weighted fluid-attenuated inversion recovery; T1CE, T1-weighted contrast-enhanced; DCE-MRI, dynamic contrast-enhanced magnetic resonance imaging; rs-Bold-fMRI, resting state-blood oxygen level dependent-functional magnetic resonance imaging; TR, repetition time; TE, echo time; FA, flip angle; FOV, field of view.

| **Tool** | **Acronym** | **Version** | **Relationship to FAE** | **Use in this study** |
| --- | --- | --- | --- | --- |
| FeAture Explorer | FAE | v0.6.0 | Core platform | Radiomic feature extraction, preprocessing, feature selection, classifier development, and performance evaluation |
| nnFeature Explorer | nnFAE | v0.2.5 | Extended module of FAE | Peritumoral VOI expansion and voxel-wise habitat partitioning |

**Table S2. Overview of FAE and nnFAE tools used in this study**

*Abbreviations*: FAE, feature explorer; nnFAE, nnfeature explorer; IBSI, image biomarker standardisation initiative.

| **Characteristics** | **Training cohort (n = 92)** | | ***z*/t**  **value** | ***p***  **value** | **Test cohort (n = 40)** | | ***z*/t**  **value** | ***p***  **value** |
| --- | --- | --- | --- | --- | --- | --- | --- | --- |
|  | **IDH-mutant**  **(n = 36)** | **IDH-wildtype**  **(n = 56)** |  |  | **IDH-mutant**  **(n = 16)** | **IDH-wildtype**  **(n = 24)** |  |  |
| **Age, years (IQR**) | 49.50 (15.00) | 61.00 (14.00) | -4.207 | < 0.001* | 49.00 (20.75) | 66.00 (21.25) | -2.834 | 0.005* |
| **Gender, n (%)** |  |  |  |  |  |  |  |  |
| Male | 18.00 (19.56) | 19.00 (20.65) | 1.526 | 0.135 | 9.00 (22.50) | 17.00 (42.50) | 0.935 | 0.500 |
| Female | 18.00 (19.56) | 37.00 (40.22) |  |  | 7.00 (17.50) | 7.00 (17.50) |  |  |
| **Tumor type, n (%)** |  |  |  |  |  |  |  |  |
| Astrocytoma | 26.00 (28.26) | 0 (0.00) | - | - | 7.00 (17.50) | 0 (0.00) | - | - |
| Oligodendroglioma | 10.00 (10.87) | 0 (0.00) |  |  | 9.00 (22.50) | 0 (0.00) |  |  |
| Glioblastoma | 0 (0.00) | 56.00 (60.87) |  |  | 0 (0.00) | 24.00 (60.00) |  |  |
| **Tumor location, n (%)** |  |  |  |  |  |  |  |  |
| Frontal lobe | 18.00 (19.56) | 15.00 (16.30) | - | - | 8.00 (20.00) | 6.00 (15.00) | - | - |
| Temporal lobe | 6.00 (6.52) | 25.00 (27.17) |  |  | 2.00 (5.00) | 8.00 (20.00) |  |  |
| Parietal lobe | 7.00 (7.61) | 8.00 (8.70) |  |  | 2.00 (5.00) | 4.00 (10.00) |  |  |
| Occipital lobe | 1.00 (1.08) | 3.00 (3.26) |  |  | 1.00 (2.50) | 3.00 (7.50) |  |  |
| Multilobar lobes | 4.00 (4.34) | 5.00 (5.43) |  |  | 3.00 (7.50) | 3.00 (7.50) |  |  |
| **Peritumoral edema, n (%)** |  |  |  |  |  |  |  |  |
| Yes | 10.00 (10.87) | 49.00 (53.26) | -5.797 | < 0.001* | 7.00 (17.50) | 23.00 (57.50) | -3.680 | < 0.001* |
| No | 26.00 (28.26) | 7.00 (7.61) |  |  | 9.00 (22.50) | 1.00 (2.50) |  |  |
| **WHO grade, n (%)** |  |  |  |  |  |  |  |  |
| 2 | 24.00 (26.09) | 0 (0.00) | -8.308 | < 0.001* | 5.00 (12.50) | 0 (0.00) | -5.141 | < 0.001* |
| 3 | 8.00 (8.70) | 0 (0.00) |  |  | 9.00 (22.50) | 0 (0.00) |  |  |
| 4 | 4.00 (4.35) | 56.00 (60.87) |  |  | 2.00 (5.00) | 24.00 (60.00) |  |  |
| **1p/19q status, n (%)** |  |  |  |  |  |  |  |  |
| Codeleted | 7.00 (7.61) | 2.00 (2.17) | 2.655 | 0.016* | 3.00 (7.50) | 0 (0.00) | 2.708 | 0.018* |
| Non-codeleted | 8.00 (8.70) | 21.00 (22.83) |  |  | 1.00 (2.50) | 8.00 (20.00) |  |  |
| NA | 21.00 (22.83) | 33.00 (35.87) |  |  | 12.00 (30.00) | 16.00 (40.00) |  |  |
| **MGMT status, n (%)** |  |  |  |  |  |  |  |  |
| Methylated | 14.00 (15.22) | 11.00 (11.96) | 2.852 | 0.005* | 4.00 (10.00) | 4.00 (10.00) | 1.658 | 0.208 |
| Unmethylated | 1.00 (1.08) | 12.00 (13.04) |  |  | 0 (0.00) | 4.00 (10.00) |  |  |
| NA | 21.00 (22.83) | 33.00 (35.87) |  |  | 12.00 (30.00) | 16.00 (40.00) |  |  |
| **TERT status, n (%)** |  |  |  |  |  |  |  |  |
| Mutant | 7.00 (7.61) | 18.00 (19.56) | -1.980 | 0.079 | 3.00 (7.50) | 5.00 (12.50) | 0.415 | 1.000 |
| Wildtype | 8.00 (8.70) | 5.00 (5.43) |  |  | 1.00 (2.50) | 3.00 (7.50) |  |  |
| NA | 21.00 (22.83) | 33.00 (35.87) |  |  | 12.00 (30.00) | 16 (40.00) |  |  |
| **EGFR status, n (%)** |  |  |  |  |  |  |  |  |
| Amplification | 2.00 (2.17) | 16.00 (17.39) | -3.348 | 0.001* | 1.00 (2.50) | 4.00 (10.00) | -0.793 | 0.576 |
| Non-amplification | 13.00 (14.13) | 7.00 (7.61) |  |  | 3.00 (7.50) | 4.00 (10.00) |  |  |
| NA | 21.00 (22.83) | 33.00 (35.87) |  |  | 12.00 (30.00) | 16.00 (40.00) |  |  |
| **Ki-67, % (IQR)** | 10.00 (22.50) | 40.00 (20.00) | -5.735 | < 0.001* | 10.00 (22.50) | 30.00 (10.00) | -3.751 | < 0.001* |
| **MVD, vessels/HPF** | 38.90 (39.12) | 20.24 (15.68) | -4.42 | < 0.001* | 27.44 ± 11.71 | 54.25 ± 32.08 | 3.193 | 0.003* |

**Table S3. Demographic and clinical characteristics by training and test cohort**

*Note*: Data are presented as mean ± SD or median (IQR), or n (%) as appropriate. A |z| > 1.96 corresponds to p < 0.05. *, represents statistical significance at p < 0.05.

*Abbreviations*: SD, standard deviation; IQR, interquartile range; WHO, world health organization; IDH, isocitrate dehydrogenase; NA, no molecular results; MGMT, O6-methylguanine-DNA methyltransferase; TERT, telomerase reverse transcriptase; EGFR, epidermal growth factor receptor; MVD, microvessel density (vessels/HPF); HPF, high-power field (×400).

| **Radiomic models/**  **Feature count** | **AUC** | **95% CI** | **Sensitivity** | **Specificity** | **ACC** | **PPV** | **NPV** | **Youden** | **Cohorts** |
| --- | --- | --- | --- | --- | --- | --- | --- | --- | --- |
| **Intratumoral models/Feature count** | |  |  |  |  |  |  |  |  |
| Whole-tumor (24) | 0.954 | 0.912 - 0.997 | 0.9720 | 0.929 | 0.946 | 0.897 | 0.981 | 0.901 | Training |
|  | 0.864 | 0.783 - 0.945 | 0.889 | 0.821 | 0.848 | 0.762 | 0.920 | 0.710 | Validation |
|  | 0.753 | 0.589 - 0.917 | 0.500 | 0.958 | 0.775 | 0.889 | 0.742 | 0.458 | Test |
| **Habitat* (13)** | **0.945** | 0.901 - 0.989 | 0.861 | 0.929 | 0.902 | 0.886 | 0.912 | 0.790 | Training |
|  | **0.868** | 0.789 - 0.946 | 0.806 | 0.911 | 0.870 | 0.853 | 0.879 | 0.716 | Validation |
|  | **0.854** | 0.721 - 0.988 | 0.813 | 0.917 | 0.875 | 0.867 | 0.880 | 0.729 | Test |
| Subregion1 (4) | 0.860 | 0.772 - 0.947 | 0.667 | 0.964 | 0.848 | 0.923 | 0.818 | 0.631 | Training |
|  | 0.845 | 0.752 - 0.938 | 0.667 | 0.982 | 0.859 | 0.960 | 0.821 | 0.649 | Validation |
|  | 0.792 | 0.621 - 0.962 | 0.813 | 0.833 | 0.825 | 0.765 | 0.870 | 0.646 | Test |
| Subregion2 (8) | 0.942 | 0.894 - 0.990 | 0.889 | 0.875 | 0.880 | 0.821 | 0.924 | 0.764 | Training |
|  | 0.879 | 0.798 - 0.961 | 0.889 | 0.857 | 0.870 | 0.800 | 0.923 | 0.746 | Validation |
|  | 0.841 | 0.714 - 0.969 | 0.875 | 0.708 | 0.775 | 0.667 | 0.895 | 0.583 | Test |
| Subregion3 (26) | 0.953 | 0.912 - 0.994 | 0.944 | 0.893 | 0.913 | 0.850 | 0.962 | 0.837 | Training |
|  | 0.862 | 0.777 - 0.948 | 0.833 | 0.786 | 0.804 | 0.714 | 0.880 | 0.619 | Validation |
|  | 0.818 | 0.663 - 0.973 | 0.688 | 0.958 | 0.850 | 0.917 | 0.821 | 0.646 | Test |
| **Peritumoral model/Feature count** | |  |  |  |  |  |  |  | Validation |
| Peri10mm (7) | 0.871 | 0.796 - 0.945 | 0.833 | 0.839 | 0.837 | 0.769 | 0.887 | 0.673 | Training |
|  | 0.847 | 0.765 - 0.928 | 0.722 | 0.875 | 0.815 | 0.788 | 0.831 | 0.597 | Validation |
|  | 0.776 | 0.614 - 0.938 | 0.750 | 0.833 | 0.800 | 0.750 | 0.833 | 0.583 | Test |
| **Combined models/Feature count** | | |  |  |  |  |  |  | Validation |
| Whole-tumor - Peri10mm (14) | 0.967 | 0.927 - 1.000 | 0.944 | 0.946 | 0.946 | 0.919 | 0.964 | 0.891 | Training |
|  | 0.920 | 0.856 - 0.984 | 0.917 | 0.839 | 0.870 | 0.786 | 0.940 | 0.756 | Validation |
|  | 0.867 | 0.758 - 0.976 | 0.813 | 0.833 | 0.825 | 0.765 | 0.870 | 0.646 | Test |
| **Habitat - Peri10mm* (22)** | **0.967** | 0.934 - 1.000 | 0.944 | 0.946 | 0.946 | 0.919 | 0.964 | 0.891 | Training |
|  | **0.890** | 0.820 - 0.961 | 0.806 | 0.893 | 0.859 | 0.829 | 0.877 | 0.698 | Validation |
|  | **0.914** | 0.831 - 0.997 | 0.938 | 0.750 | 0.825 | 0.714 | 0.947 | 0.688 | Test |
| Subregion1 - Peri10mm (10) | 0.930 | 0.872 - 0.987 | 0.806 | 0.929 | 0.880 | 0.879 | 0.881 | 0.734 | Training |
|  | 0.903 | 0.840 - 0.966 | 0.833 | 0.875 | 0.859 | 0.811 | 0.891 | 0.708 | Validation |
|  | 0.823 | 0.690 - 0.956 | 0.750 | 0.792 | 0.775 | 0.706 | 0.826 | 0.542 | Test |
| Subregion2 - Peri10mm (15) | 0.953 | 0.908 - 0.999 | 0.917 | 0.911 | 0.913 | 0.868 | 0.944 | 0.827 | Training |
|  | 0.881 | 0.799 - 0.964 | 0.806 | 0.946 | 0.891 | 0.906 | 0.883 | 0.752 | Validation |
|  | 0.885 | 0.755 - 1.000 | 0.938 | 0.792 | 0.850 | 0.750 | 0.950 | 0.729 | Test |
| Subregion3 - Peri10mm (12) | 0.963 | 0.930 - 0.997 | 0.889 | 0.946 | 0.924 | 0.914 | 0.930 | 0.835 | Training |
|  | 0.907 | 0.841 - 0.973 | 0.833 | 0.911 | 0.880 | 0.857 | 0.895 | 0.744 | Validation |
|  | 0.839 | 0.694 - 0.984 | 0.688 | 0.917 | 0.825 | 0.846 | 0.815 | 0.604 | Test |

**Table S4. Performance of intratumoral and peritumoral radiomic models based on *K*^trans^ - *r*CVR**

*Note*: Bold with an asterisk (*) means the optimal radiomic model based on DCE-derived *K*^trans^ and BOLD-derived *r*CVR.

*Abbreviations*: AUC, area under the curve; 95% CI, 95% confidence interval; ACC, accuracy; PPV, positive predictive value; NPV, negative predictive value; DCE-MRI, dynamic contrast-enhanced magnetic resonance imaging; rs-Bold-fMRI, resting state-blood oxygen level dependent-functional magnetic resonance imaging; *K*^trans^, the volume transfer constant; *r*CVR, relative cerebrovascular reactivity; Peri10mm, peritumoral 10mm region.

| **Radiomic models** | **NRI** | **95% CI** | **SE** | ***z* value** | ***p* value** | **IDI** | **95% CI** | **SE** | ***z* value** | ***p* value** |
| --- | --- | --- | --- | --- | --- | --- | --- | --- | --- | --- |
| **Whole-tumor *vs.* Habitat** |  |  |  |  |  |  |  |  |  |  |
| Training | -0.056 | -0.178 - 0.067 | 0.063 | -0.886 | 0.375 | -0.031 | -0.113 - 0.050 | 0.042 | -0.754 | 0.451 |
| Test | 0.396 | 0.132 - 0.660 | 0.135 | 2.940 | 0.003* | 0.125 | -0.059 - 0.308 | 0.094 | 1.333 | 0.183 |
| **Habitat *vs.* Habitat-Peri10mm** |  |  |  |  |  |  |  |  |  |  |
| Training | 0.064 | -0.039 - 0.166 | 0.052 | 1.214 | 0.225 | 0.034 | -0.010 - 0.078 | 0.022 | 1.528 | 0.126 |
| Test | -0.250 | -0.459 - -0.041 | 0.107 | -2.342 | 0.019* | 0.040 | -0.057 - 0.136 | 0.049 | 0.801 | 0.423 |
| **Peri10mm *vs.* Habitat-Peri10mm** |  |  |  |  |  |  |  |  |  |  |
| Training | 0.873 | 0.497 - 1.249 | 0.192 | 4.547 | < 0.001* | 0.202 | 0.118 - 0.285 | 0.042 | 4.748 | < 0.001* |
| Test | 0.792 | 0.221 - 1.362 | 0.291 | 2.721 | 0.007* | 0.212 | 0.028 - 0.396 | 0.094 | 2.262 | 0.024* |
| **Whole-tumor *vs.* Habitat-Peri10mm** |  |  |  |  |  |  |  |  |  |  |
| Training | 0.060 | -0.355 - 0.474 | 0.211 | 0.282 | 0.778 | 0.003 | -0.079 - 0.084 | 0.042 | 0.067 | 0.946 |
| Test | 0.354 | 0.005 - 0.703 | 0.178 | 1.990 | 0.046* | 0.164 | -0.055 - 0.381 | 0.112 | 1.468 | 0.142 |

**Table S5. Performance comparison of DCE and Bold-based radiomic model with NRI and IDI**

*Note*: *, represents statistical significance at p < 0.05.

*Abbreviations*: DCE-MRI, dynamic contrast-enhanced magnetic resonance imaging; rs-Bold-fMRI, resting state-blood oxygen level dependent- functional magnetic resonance imaging; NRI, net reclassification improvement; IDI, integrated discrimination improvement; SE, standard error; 95% CI, 95% confidence interval; Peri10mm, peritumoral 10mm region.

| **Mean values of parameters** | **Man Whitney *U* test/**  **Two-sample independent *t* test** | | ***z/t* value** | ***p* value** |
| --- | --- | --- | --- | --- |
|  | **IDH-wildtype**  **(n = 80)** | **IDH-mutant**  **(n = 52)** |  |  |
| ***K*^trans^ (min^−1^)** |  |  |  |  |
| Subregion1 | 12.43 ± 3.13 | 11.89 ± 3.14 | 0.975 | 0.331 |
| Subregion2 | 72.73 (9.62) | 48.30 (13.80) | -6.189 | < 0.001* |
| Subregion3 | 272.10 (67.28) | 269.26 (151.76) | -0.088 | 0.929 |
| Peri10mm | 28.54 (17.87) | 26.10 (16.36) | -1.425 | 0.154 |
| ***r*CVR** |  |  |  |  |
| Subregion1 | 0.42 (0.62) | 0.43 (0.58) | -0.782 | 0.434 |
| Subregion2 | 0.43 (0.60) | 0.53 (0.73) | -1.886 | 0.059 |
| Subregion3 | 0.40 (0.74) | 0.46 (0.71) | -0.675 | 0.499 |
| Peri10mm | 0.66 (0.37) | 0.76 (0.36) | -1.965 | 0.049* |
| **Radscore** | 2.10 ± 1.59 | -2.67 ± 2.54 | 13.265 | < 0.001* |
| **ITH score** | 0.79 (0.21) | 0.40 (0.39) | -6.268 | < 0.001* |

**Table S6 Comparisons of regional *K*^trans^ and *r*CVR values and Radscore between IDH-mutant and IDH-wildtype gliomas**

*Note*: Data are presented as mean ± SD for normally distributed variables, or as median (IQR) for non-normally distributed variables. n, represents sample size. A |z| > 1.96 corresponds to p < 0.05. *, represents statistical significance at p < 0.05.

*Abbreviations*: SD, standard deviation; IQR, interquartile range; IDH, isocitrate dehydrogenase; *K*^trans^, the volume transfer constant; *r*CVR, relative cerebrovascular reactivity; peri10mm, peritumoral 10mm region; Radscore, radiomic score; ITH, intratumoral heterogeneity.

| **Mean values of parameters** | **One-way ANOVA/Kruskal-Wallis test** | | | **Test statistic** | ***p* value** |
| --- | --- | --- | --- | --- | --- |
|  | **IDH-mutant (n = 52)** | | |  |  |
| ***K*^trans^ (min^−1^)** | **WHO grade2**  **(n = 29)** | **WHO grade 3**  **(n = 17)** | **WHO grade 4**  **(n = 6)** |  |  |
| Subregion1 | 11.82 ± 3.00 | 11.24 ± 3.20 | 14.08 ± 3.21 | 1.896 | 0.161 |
| Subregion2 | 46.14 (12.55) | 47.99 (13.13) | 77.67 (22.50) | 10.236 | 0.006* |
| Subregion3 | 243.44 (120.02) | 290.85 (134.91) | 274.98 (174.66) | 2.844 | 0.241 |
| Peri10mm | 26.94 (16.69) | 25.26 (14.83) | 26.39 (22.88) | 0.537 | 0.765 |
| ***r*CVR** |  |  |  |  |  |
| Subregion1 | 0.44 (0.62) | 0.40 (0.92) | 0.44 (0.44) | 0.032 | 0.984 |
| Subregion2 | 0.57 (0.57) | 0.50 (0.92) | 0.37 (0.62) | 1.595 | 0.450 |
| Subregion3 | 0.52 ± 0.49 | 0.71 ± 0.61 | 0.38 ± 0.38 | 1.213 | 0.306 |
| Peri10mm | 0.81 ± 0.29 | 0.85 ± 0.26 | 0.64 ± 0.12 | 1.486 | 0.236 |

**Table S7. Within-group comparisons of imaging parameters among IDH-mutant cases stratified by WHO grade**

*Note*: Data are presented as mean ± SD for normally distributed variables, or as median (IQR) for non-normally distributed variables. n, represents sample size. *, represents statistical significance at p < 0.05.

*Abbreviations*: SD, standard deviation; IQR, interquartile range; IDH, isocitrate dehydrogenase; WHO, world health organization; ANOVA, analysis of variance; *K*^trans^, the volume transfer constant; *r*CVR, relative cerebrovascular reactivity; peri10mm, peritumoral 10mm region.

| **Optimal radiomic features (22)** | **Mann Whitney *U* test/**  **Two-sample independent *t* test** | | ***z/t* value** | ***p* value** | **FDR**  **correction** |
| --- | --- | --- | --- | --- | --- |
|  | **Edema**  **(n = 89)** | **No edema**  **(n = 43)** |  |  |  |
| S1 *K*^trans^ glszm SmallAreaEmphasis | 0.75 ± 0.05 | 0.70 ± 0.04 | -6.350 | < 0.001* | 0.015* |
| S2 shape Flatness | 0.63 ± 0.13 | 0.53 ± 0.15 | -3.840 | < 0.001* | 0.04* |
| S2 *K*^trans^ firstorder Entropy | 3.80 (0.32) | 2.96 (0.64) | -6.043 | < 0.001* | 0.02* |
| S2 *K*^trans^ glrlm GrayLevelVariance | 18.09 (5.70) | 10.67 (5.13) | -4.771 | < 0.001* | 0.0325* |
| S2 *K*^trans^ glrlm LongRunHighGrayLevelEmphasis | 67.99 (28.21) | 31.28 (27.44) | -5.742 | < 0.001* | 0.0225* |
| S2 *K*^trans^ glszm GrayLevelVariance | 19.03 (3.41) | 13.71 (4.86) | -5.358 | < 0.001* | 0.0275* |
| S2 *K*^trans^ glszm SmallAreaHighGrayLevelEmphasis | 57.64 (13.68) | 36.06 (20.56) | -6.441 | < 0.001* | 0.0125* |
| S2 *K*^trans^ ngtdm Busyness | 12.08 (11.72) | 3.16 (6.50) | -5.620 | < 0.001* | 0.025* |
| S2 *r*CVR glszm SmallAreaEmphasis | 0.52 ± 0.06 | 0.61 ± 0.12 | 5.276 | < 0.001* | 0.03* |
| S3 shape LeastAxisLength | 26.69 ± 8.99 | 14.17 ± 11.01 | -6.960 | < 0.001* | 0.0025* |
| S3 *K*^trans^ firstorder MeanAbsoluteDeviation | 95.43 (67.08) | 76.40 (129.01) | -1.190 | 0.234 | - |
| S3 *K*^trans^ firstorder Variance | 28019.01 (39642.03) | 12739.45 (61173.27) | -1.632 | 0.103 | - |
| S3 *r*CVR firstorder Kurtosis | 3.29 (1.92) | 2.57 (1.38) | -3.472 | 0.001* | 0.0425* |
| S3 *K*^trans^ gldm DependenceNonUniformity | 79.74 (82.78) | 13.27 (28.73) | -6.793 | < 0.001* | 0.0075* |
| S3 *K*^trans^ glrlm RunLengthNonUniformity | 433.45 (522.45) | 24.03 (102.99) | -6.893 | < 0.001* | 0.005* |
| S3 *K*^trans^ glszm SizeZoneNonUniformity | 61.36 (50.18) | 13.47 (27.36) | -6.215 | < 0.001* | 0.0175* |
| Peri10mm shape Elongation | 0.77(0.13) | 0.77 (0.18) | -0.755 | 0.450 | - |
| Peri10mm shape Flatness | 0.51 ± 0.11 | 0.43 ± 0.13 | -3.857 | < 0.001* | 0.0375* |
| Peri10mm shape LeastAxisLength | 42.72 ± 10.20 | 34.16 ± 12.55 | -4.185 | < 0.001* | 0.035* |
| Peri10mm *r*CVR firstorder Maximum | 2.59 (1.48) | 3.08 (1.35) | -2.119 | 0.034* | 0.05* |
| Peri10mm *K*^trans^ glcm Correlation | 0.15 (0.08) | 0.13 (0.05) | -2.153 | 0.031* | 0.0475* |
| Peri10mm *K*^trans^ glcm Imc1 | -0.07 (0.03) | -0.06 (0.03) | -2.251 | 0.024* | 0.045* |
| Radscore | 1.59 (1.86) | -2.90 (4.82) | 6.465 | < 0.001* | 0.01* |

**Table S8. Comparison of optimal radiomic features between edema-positive and edema-negative groups using the DCE-BOLD-driven Habitat-Peri10mm model**

*Note*: Data are presented as mean ± SD, median (IQR). n, represents sample size. A |z| > 1.96 corresponds to p < 0.05. *, represents statistical significance at p < 0.05. To control the FDR, p values were adjusted using the Benjamini-Hochberg procedure with a threshold of α < 0.05. *Abbreviations*: SD, standard deviation; IQR, interquartile range; FDR, false discovery rate; DCE-MRI, dynamic contrast-enhanced magnetic resonance imaging; rs-BOLD-fMRI, resting state-blood oxygen level dependent-functional magnetic resonance imaging; *K*^trans^, volume transfer constant; *r*CVR, relative cerebrovascular reactivity; S1, subregion1; S2, subregion2; S3, subregion3; peri10mm, peritumoral 10mm region; glszm, gray-level size zone matrix; glcm, gray-level co-occurrence matrix; ngtdm, neighborhood gray-tone difference matrix; gldm, gray-level dependence matrix, glrlm, gray level run length matrix; Imc1, information measure of correlation1; Radscore, radiomic score.

| **ITH score** | **Two-sample independent *t* test** | | | | | | | |
| --- | --- | --- | --- | --- | --- | --- | --- | --- |
| **ITH score** | **IDH-wildtype**  **(n = 31)** | | ***t* value** | ***p* value** | **IDH-mutant**  **(n = 19)** | | ***t* value** | ***p* value** |
|  | **EGFR-**  **amplification**  **(n = 20)** | **EGFR-**  **non-amplification**  **(n = 11)** |  |  | **EGFR-**  **amplification**  **(n = 3)** | **EGFR-**  **non-amplification**  **(n = 16)** |  |  |
|  | 0.76 ± 0.14 | 0.76 ± 0.15 | -0.004 | 0.997 | 0.29 ± 0.09 | 0.45 ± 0.23 | 1.140 | 0.270 |
| **ITH score** | **EGFR-amplification**  **(n = 23)** | |  |  | **EGFR-nonamplification**  **(n = 27)** | |  |  |
|  | **IDH-wildtype**  **(n = 20)** | **IDH-mutant**  **(n = 3)** |  |  | **IDH-wildtype**  **(n = 11)** | **IDH-mutant**  **(n = 16)** |  |  |
|  | 0.76 ± 0.15 | 0.29 ± 0.09 | 5.192 | < 0.001* | 0.76 ± 0.14 | 0.45 ± 0.23 | 3.987 | 0.001* |

**Table S9. Comparison of ITH score by EGFR status, stratified by IDH mutation status**

*Note*: Data are presented as mean ± SD. n, represents sample size. *, represents statistical significance at p < 0.05.

*Abbreviations*: SD, standard deviation; ITH, intratumoral heterogeneity; IDH, isocitrate dehydrogenase; EGFR, epidermal growth factor receptor.

| **Variables** | **Univariable analysis** | | **Multivariable analysis** | |
| --- | --- | --- | --- | --- |
|  | **HR (95% CI)** | ***p* value^1^** | **HR (95% CI)** | ***p* value^2^** |
| **Clinical characteristics** |  |  |  |  |
| Age | 1.09 (1.04 - 1.13) | < 0.001* |  |  |
| Sex |  |  |  |  |
| *male* | Reference | - |  |  |
| *female* | 0.59 (0.27 - 1.30) | 0.19 |  |  |
| Peritumoral edema |  |  |  |  |
| *no* | Reference | - |  |  |
| *yes* | 3.17 (1.10 - 9.16) | 0.03* | 1.12 (0.28 - 4.51) | 0.88 |
| Chemoradiotherapy |  |  |  |  |
| *no* | Reference | - |  |  |
| *yes* | 1.20 (0.49 - 3.00) | 0.688 |  |  |
| WHO grade |  |  |  |  |
| 2 | Reference | - |  |  |
| 3 | < 0.001 | 0.979 |  |  |
| 4 | 16.68 (2.26 - 123.14) | 0.006*^+^ |  |  |
| IDH status |  |  |  |  |
| *mutant* | Reference | - |  |  |
| *wild-type* | 27.51 (3.73 - 202.99) | 0.001*^+^ |  |  |
| Ki-67 (%) | 1.03 (1.01 - 1.04) | 0.001* |  |  |
| MVD (vessels/HPF) | 1.01 (0.99 - 1.02) | 0.18 |  |  |
| **Image characteristics** |  |  |  |  |
| Radscore | 1.38 (1.18 - 1.62) | < 0.001*^+^ |  |  |
| ITH score | 15.06 (2.56 - 88.55) | 0.003* | 13.30 (1.29 - 137.19) | 0.03* |

**Table S10. Univariable and multivariable Cox regression analysis for PFS in the primary cohort**

*Note*: p value 1 for univariable hazard ratio. p value 2 for multivariable hazard ratio. p value^1^*, represents statistical significance at p < 0.10. p value^2^*, represents statistical significance at p < 0.05. +, VIF ≥ 5 indicates serious multicollinearity.

*Abbreviations*: PFS, progression-free survival; HR, hazard ratio; 95% CI, 95% confidence interval; IDH, isocitrate dehydrogenase; MVD, microvessel density (vessels/HPF); HPF, high-power field (×400); Radscore, radiomic score; ITH, intratumoral heterogeneity; VIF, variance inflation factor.

| **Variables** | ***t* value** | ***p* value** | **Collinearity statistics** | |
| --- | --- | --- | --- | --- |
|  |  |  | **Tolerance** | **VIF** |
| Age | 2.446 | 0.018 | 0.684 | 1.461* |
| Peritumoral edema | -0.680 | 0.499 | 0.378 | 2.642* |
| WHO grade | 0.196 | 0.845 | 0.143 | 6.983 |
| IDH status | -2.035 | 0.046 | 0.152 | 6.585 |
| Ki-67 | -0.770 | 0.444 | 0.405 | 2.466* |
| Radscore | 1.775 | 0.081 | 0.212 | 4.708 |
| ITH score | -0.809 | 0.422 | 0.338 | 2.963* |

**Table S11. Multicollinearity diagnostics analysis**

*Note*: *, VIF < 5 indicates no serious multicollinearity.

*Abbreviations*: VIF, variance inflation factor; IDH, isocitrate dehydrogenase; WHO, world health organization; MVD, microvessel density; Radscore, radiomic score; ITH, intratumoral heterogeneity.

| **Variables** | **Univariable analysis** | | **Multivariable analysis** | |
| --- | --- | --- | --- | --- |
|  | **HR (95% CI)** | ***p* value^1^** | **HR (95% CI)** | ***p* value^2^** |
| Combination 1 |  |  |  |  |
| **Ki-67 (%)** | 1.03 (1.01 - 1.04) | 0.001* | **1.02 (0.99 - 1.03)** | **0.13** |
| **ITH score** | 15.06 (2.56 - 88.55) | 0.003* | **7.13 (0.93 - 54.19)** | **0.06** |
| Combination 2 |  |  |  |  |
| *Age* | 1.09 (1.04 - 1.13) | < 0.001* | *1.07 (1.02 - 1.12)* | *0.01** |
| *ITH score* | 15.06 (2.56 - 88.55) | 0.003* | *4.35 (0.60 - 31.65)* | *0.15* |

**Table S12. Univariable and multivariable Cox regression analysis for PFS in the primary cohort**

*Note*: p value 1 for univariable hazard ratio. p value 2 for multivariable hazard ratio. p value^1^*, represents statistical significance at p < 0.10. p value^2^*, represents statistical significance at p < 0.05. Variables in **bold** were entered together into one multivariate Cox regression model; variables in *italics* were entered together into a separate model.

*Abbreviations*: PFS, progression-free survival; HR, hazard ratio; 95% CI, 95% confidence interval; ITH, intratumoral heterogeneity.

| **Radiomic models** | **AUC** | **95% CI** | **Sensitivity** | **Specificity** | **ACC** | **PPV** | **NPV** | **Youden** |
| --- | --- | --- | --- | --- | --- | --- | --- | --- |
| **Intratumoral models** | |  |  |  |  |  |  |  |
| Whole-tumor | 0.840 | 0.743-0.938 | 0.806 | 0.875 | 0.848 | 0.806 | 0.875 | 0.400 |
| Habitat | 0.920 | 0.855-0.977 | 0.861 | 0.911 | 0.891 | 0.861 | 0.911 | 0.441 |
| Subregion1 | 0.872 | 0.792-0.952 | 0.722 | 0.929 | 0.848 | 0.867 | 0.839 | 0.553 |
| Subregion2 | 0.919 | 0.866-0.972 | 0.889 | 0.839 | 0.859 | 0.780 | 0.922 | 0.356 |
| Subregion3 | 0.822 | 0.726-0.919 | 0.806 | 0.804 | 0.804 | 0.725 | 0.865 | 0.422 |
| **Peritumoral model** | |  |  |  |  |  |  |  |
| Peri10mm | 0.796 | 0.703-0.888 | 0.750 | 0.786 | 0.772 | 0.692 | 0.830 | 0.426 |
| **Combined models** | |  |  |  |  |  |  |  |
| Whole-tumor -Peri10mm | 0.870 | 0.792-0.948 | 0.806 | 0.875 | 0.848 | 0.806 | 0.875 | 0.502 |
| Habitat - Peri10mm | 0.858 | 0.768-0.948 | 0.806 | 0.911 | 0.870 | 0.853 | 0.879 | 0.412 |
| Subregion1 - Peri10mm | 0.849 | 0.765-0.932 | 0.750 | 0.857 | 0.815 | 0.771 | 0.842 | 0.453 |
| Subregion2 - Peri10mm | 0.877 | 0.795-0.960 | 0.917 | 0.804 | 0.848 | 0.750 | 0.938 | 0.308 |
| Subregion3 - Peri10mm | 0.873 | 0.793-0.953 | 0.750 | 0.929 | 0.859 | 0.871 | 0.852 | 0.540 |

**Table S13. Leave-one-out cross-validation performance of radiomic models in the training cohort**

*Note*: Performance was evaluated using LOOCV within the training cohort (n = 92). AUCs were calculated from pooled held-out predictions, and 95% confidence intervals were estimated using 2,000 stratified bootstrap resamples.

*Abbreviations*: LOOCV, leave-one-out cross-validation; AUC, area under the receiver operating characteristic curve; CI, confidence interval; ACC, accuracy; PPV, positive predictive value; NPV, negative predictive value; Peri10mm, 10-mm peritumoral region.

**Supplementary Fig. S1** - **Study participant screening and enrollment flowchart**

*Note*: n, represents sample size.

*Abbreviations*: IDH, isocitrate dehydrogenase; T1WI, T1-weighted imaging; T2WI, T2-weighted imaging; T2-FLAIR, T2-weighted fluid-attenuated inversion recovery; T1CE, T1-weighted contrast enhanced imaging; MRI, magnetic resonance imaging; rs-Bold-fMRI, resting state-blood oxygen level dependent-functional MRI; DCE-MRI, dynamic contrast-enhanced MRI; WHO, world health organization.

**Supplementary Fig. S2** - **DeLong's test between the intratumoral and peritumoral radiomic models in the training cohort (a)and test cohort (b)**

*Note*: The numerical values in the heatmap represent p values. The color bar indicates p values.

*Abbreviation*: Peri10mm, peritumoral 10mm region.

**Supplementary Fig. S3** - **Development of Radscore for the optimal Habitat-Peri10mm radiomic model**

*Abbreviations*: Radscore, radiomic score; *K*^trans^, volume transfer constant; *r*CVR, relative cerebrovascular reactivity; S1, subregion1; S2, subregion2; S3, subregion3; peri10mm, peritumoral 10mm region; glszm, gray-level size zone matrix; glcm, gray-level co-occurrence matrix; ngtdm, neighborhood gray-tone difference matrix; gldm, gray-level dependence matrix; glrlm, gray level run length matrix; Imc1, information measure of correlation1; IDH, isocitrate dehydrogenase.

**Supplementary Fig. S4** - **Spearman correlation matrix between key radiomic features and the Ki-67 index and MVD**

*Note*: *****, p<0.05; **, p<0.01; ***, p<0.001. The color bar represents r value.

*Abbreviations*: *K*^trans^, volume transfer constant; *r*CVR, relative cerebrovascular reactivity; MVD, microvessel density; S1, subregion1; S2, subregion2; S3, subregion3; peri10mm, peritumoral 10mm region; glszm, gray-level size zone matrix; glcm, gray-level co-occurrence matrix; ngtdm, neighborhood gray-tone difference matrix; gldm, gray-level dependence matrix; glrlm, gray level run length matrix; Imc1, information measure of correlation1.

**Supplementary Fig. S5** - **Quantification of ITH using PCA**

*Abbreviations*: PCA, principal component analysis; ITH, intratumoral heterogeneity; Dim, dimension; Contrib, contribution.

**Supplementary Fig. S6 - Exploratory PFS Kaplan‑Meier curves (a) and multivariable Cox forest plot (b), and correlations of ITH score with HIF-1α (c) and VEGF-A (d) expression**

*Abbreviations*: ITH, intratumoral heterogeneity; HIF-1α, hypoxia-inducible factor-1alpha; VEGF-A, vascular endothelial growth factor A; PFS, progression-free survival.
